# Survey on open peer review: Attitudes and experience amongst editors, authors and reviewers

**DOI:** 10.1371/journal.pone.0189311

**Published:** 2017-12-13

**Authors:** Tony Ross-Hellauer, Arvid Deppe, Birgit Schmidt

**Affiliations:** 1 Know-Center GmbH, Graz, Austria; 2 Kassel University Library, University of Kassel, Kassel, Germany; 3 State and University Library Goettingen, University of Goettingen, Goettingen, Germany; Tilburg University, NETHERLANDS

## Abstract

Open peer review (OPR) is a cornerstone of the emergent Open Science agenda. Yet to date no large-scale survey of attitudes towards OPR amongst academic editors, authors, reviewers and publishers has been undertaken. This paper presents the findings of an online survey, conducted for the OpenAIRE2020 project during September and October 2016, that sought to bridge this information gap in order to aid the development of appropriate OPR approaches by providing evidence about attitudes towards and levels of experience with OPR. The results of this cross-disciplinary survey, which received 3,062 full responses, show the majority (60.3%) of respondents to be believe that OPR as a general concept should be mainstream scholarly practice (although attitudes to individual traits varied, and open identities peer review was not generally favoured). Respondents were also in favour of other areas of Open Science, like Open Access (88.2%) and Open Data (80.3%). Among respondents we observed high levels of experience with OPR, with three out of four (76.2%) reporting having taken part in an OPR process as author, reviewer or editor. There were also high levels of support for most of the traits of OPR, particularly open interaction, open reports and final-version commenting. Respondents were against opening reviewer identities to authors, however, with more than half believing it would make peer review worse. Overall satisfaction with the peer review system used by scholarly journals seems to strongly vary across disciplines. Taken together, these findings are very encouraging for OPR’s prospects for moving mainstream but indicate that due care must be taken to avoid a “one-size fits all” solution and to tailor such systems to differing (especially disciplinary) contexts. OPR is an evolving phenomenon and hence future studies are to be encouraged, especially to further explore differences between disciplines and monitor the evolution of attitudes.

## Introduction

Traditional peer review is generally (1) anonymous, with either the reviewer unknown to the author (single-blind review) or both author and reviewer unknown to each other (double-blind review); (2) selective, with reviewers selected by editors; and (3) confidential, with neither the review process nor the reviews themselves made public. This model has long been recognized to have significant flaws, accused of being unreliable (by, e.g., failing to detect errors or demonstrating inconsistency between reviewer reports) [1–6], taking too long (i.e., delaying times between submission and publication) [7–9], being unaccountable and enabling social and publication biases [10–12], lacking in incentive for reviewers [7, 9], and being wasteful of effort (as the same manuscript may be reviewed many times as it goes through cycles of submission and rejection) [13].

In response to these criticisms, a wide variety of changes to peer review have been suggested (see the extensive overviews in [14, 15]). Amongst these innovations, many have been labelled as “open peer review” at one time or another. As we shall see, these innovations labelled as OPR in fact encompass a wide variety of discrete ways in which peer review can be “opened up”. According to the literature review in [16], open peer review (OPR) is an umbrella term for a number of overlapping ways that peer review models can be adapted in line with the ethos of Open Science, including making reviewer and author identities open, publishing review reports and enabling greater participation in the peer review process. The full list of traits is:

- **Open identities:** Authors and reviewers are aware of each other’s identity
- **Open reports:** Review reports are published alongside the relevant article.
- **Open participation:** The wider community are able to contribute to the review process.
- **Open interaction:** Direct reciprocal discussion between author(s) and reviewers, and/or between reviewers, is allowed and encouraged.
- **Open pre-review manuscripts:** Manuscripts are made immediately available (e.g., via pre-print servers like arXiv) in advance of any formal peer review procedures.
- **Open final-version commenting:** Review or commenting on final “version of record” publications.
- **Open platforms:** Review is de-coupled from publishing in that it is facilitated by a different organizational entity than the venue of publication.

These elements are often complementary, and can be combined in various ways to produce a broad continuum of ‘openness’ in OPR. Each of these distinct traits are theorized to address one or more of the shortcomings listed above, but no trait is claimed to address all of them and sometimes their aims may be in conflict. Following [16], we can summarise these proposed benefits and drawbacks thus:

- **Unreliability and inconsistency:** *Open identities* and *open reports* are theorized to lead to better reviews, as the thought of having their name publicly connected to a work or seeing their review published encourages reviewers to be more thorough. *Open participation* and *open final-version commenting* could improve the reliability of peer review by increasing the number of potential reviewers, especially from different disciplinary backgrounds (although in practice, open participation struggles to attract reviewers). Some evidence suggests that *open interaction* between reviewers and authors could lead to improved reviewing accuracy.
- **Delay and expense:** *Open pre-review manuscripts* sharply reduce the time before research is first publicly available. *Open platforms* can help overcome the “waterfall” problem, where individual articles go through multiple cycles of review and rejection at different journals. In principle, *open participation* could reduce the need for editorial mediation in finding reviewers, but in practice any reduction of costs is questionable, as open participation can fail to attract reviewers and in any case, editorial mediation will continue to be necessary to facilitate discussion and arbitrate disputes. *Open identities* and *open reports* might actually exacerbate problems of delay and expense, as it seems invited reviewers are currently less inclined to review under such circumstances. Finally, *open interaction*–by necessitating more back and forth between reviewers and authors, and more editorial mediation–might lead to longer reviewing times.
- **Lack of accountability and risks of subversion:** *Open identities* and *reports* can increase accountability through increased transparency and by making any conflicts of interest more immediately apparent to authors and future readers. *Open participation* could overcome problems associated with editorial selection of reviewers (e.g. biases, closed-networks, elitism). However, in opening up participation to the wider community, it might actually increase engagement by those with conflicts of interest. Where anonymity is possible, this may be particularly problematic. Moreover, lack of anonymity for reviewers in *open identities* review might subvert the process by discouraging reviewers from making strong criticisms, especially against higher-status colleagues.
- **Social and publication biases:** *Open reports* adds another layer of quality assurance, allowing the wider community to scrutinize reviews to examine decision-making processes. However, *open identities* removes anonymity conditions for reviewers (single-blind) or authors and reviewers (double-blind) which are traditionally in place to counteract social biases (although there is not strong-evidence that such anonymity has been effective).
- **Lack of incentives:** *Open reports* linked to *open identities* enable higher visibility for peer review activities, allowing review work to be cited in other publications and in career development activities linked to promotion and tenure. *Open participation* could in principle increase incentives to peer review by enabling reviewers to themselves select the works that they consider themselves qualified to judge; in practice, however, experience to date suggests that reviewers are less likely to review under this condition.
- **Wastefulness:** *Open reports* make currently invisible but potentially useful scholarly information available for re-use, as well as providing young researchers a guide (to tone, length, the formulation of criticisms) to help them as they begin to do peer review themselves.

The ideas behind OPR have been around since at least the mid-1970s [17], and the term itself stems from the early 1980s [18, 19]. The first implementations and trials that explicitly categorized themselves as “open peer review” emerged in the late 20th Century [20], and some variation of OPR is now the established mode of peer review for many journals and publishers [21]. Ross-Hellauer [16] analysed 122 separate definitions to find a total of 22 unique combinations of these terms in the literature on OPR. Which of these 22 proposed combinations are most preferable, in which (disciplinary) contexts and for which purposes?

A crucial part of answering these questions is to adequately gauge the views of authors, reviewers and editors involved in scholarly publishing. This unfortunately currently constitutes an information gap, with a lack of research detailing stakeholders’ attitudes towards these different systems. What are current levels of awareness of various forms of OPR? What do editors, authors and reviewers actually want? What differences exist in attitudes between disciplines? What are the barriers that stand in the way of the uptake of such systems? What are the current levels of experience amongst stakeholders with OPR? To date no systematic analysis of these questions has been undertaken.

OpenAIRE (http://openaire.eu) is a socio-technical digital infrastructure that brings together more than 50 institutions and organisations to foster the social and technical links that enable Open Science in Europe and beyond. In addition to operating an Open Access/Open Science support, outreach and advocacy network of 33 National Open Access Desks (NOADs) across Europe, OpenAIRE serves the public interest by increasing the visibility of research outputs and linking digital entities to enable navigation. OpenAIRE also undertakes a range of research and development activities to further the evolution of scholarly communications towards openness, transparency and interoperability. As part of these latter activities, between 8^th^ September and 7^th^ October 2016, OpenAIRE undertook a survey designed to aid the development of appropriate OPR approaches by providing evidence about attitudes towards and levels of experience with OPR amongst authors, editors and reviewers. We here present the findings of this research.

## Background

In the last decade, a few large-scale, largely publisher-led studies have gauged attitudes to peer review. Although none have heretofore focused fully on attitudes to OPR, they nonetheless have touched on issues germane to this study. These studies tend to show that although researchers believe peer review is necessary, there is a belief that the current model is sub-optimal. Ware’s 2008 survey for the Publishing Research Consortium (PRC) [9], for example, found that four out of five respondents (85%) agreed that “peer review greatly helps scientific communication” and that even more (around 90%) said their own last published paper had been improved by peer review. Yet while almost two thirds (64%) declared that they were satisfied with the current system of peer review, less than a third (32%) believed that this system is the best possible. A recent follow-up study by the same author reported a slight increase in the desire for improvements in peer review [22]. The same studies found that the proportion agreeing that peer review holds back scientific communication had risen a little, from 19% in 2007 to 26% in 2015), and that the proportion who believe peer review helps scholarly communication had fallen from 85% in 2007 to 75% in 2015.

Given that most studies have been undertaken by publishers, it is perhaps understandable that incentivising and motivating reviewers has been a major feature of these surveys. Across studies, scholars gave their main reason for reviewing as being part of a reciprocal critical community [9, 23, 24]. Yet in absence of financial rewards from the publishers for whom they deliver expert advice, and with no direct acknowledgment from their employing institutions, reviewers are often nonetheless reticent. Taylor & Francis’ 2015 survey found that 60% of editors have difficulty in finding qualified reviewers [24]. Relatedly, Ware [22] found that more than a quarter of respondents thought peer review unsustainable due to there being too few willing reviewers. A major reason for this is revealed by a July 2015 survey of 2,982 respondents by Wiley, which showed that reviewers strongly believed that reviewing is currently insufficiently acknowledged and should be better recognized in institutional evaluation processes, reporting that they would spend more time reviewing if this were the case [25]. Against this background, questions of whether particular innovations might encourage or inhibit reviewers to review become pertinent.

Previous studies suggest moderate but growing support for OPR. Mark Ware asked respondents to state their preferred option amongst four kinds of review, including open identities and post-publication review. At that time, he found a strong preference for double-blind review (56%), followed by single-blind (25%), with only 13% preferring open identities and 5% post-publication review. Indeed, many reviewers (47%) claimed open identities would make them less likely to review for a journal [26]. Sense About Science’s 2009 survey found similar skepticism, with just 20% of respondents believing that open identities would be effective in improving peer review, with open reports and open identities performing only a little better (25%). Responses were similar across disciplines [23].

However, Taylor & Francis Group’s 2015 survey of 7438 researchers shows stakeholders becoming a little more “open” to OPR. It found that views of open identities (“open”), open identities plus open reports (“open and published”) and open pre-review manuscripts (“post-publication review”) were quite similar, often rated between 5 and 6 out of 10. Editors in the social sciences and humanities (SSH) were less supportive than those in science, technology and medicine (STM) subjects, and editors and reviewers were less supportive than authors. Fewer than half of respondents said that publishing their reports or making identities open would incentivize them to review. The strongest deterrents were publishing the reviewer’s report, with anonymous publication only marginally less of a deterrent than publishing their named report [24].

Running an updated survey for the Publishing Research Consortium in 2015, Ware found that attitudes towards OPR were changing and that support had grown such that 50–70% of researchers were either supportive of or neutral towards open identities review, with 35–55% also holding positive/neutral opinions of also publishing review reports. Ware noted that these levels of acceptance of open identities were in line with the experiences of journals offering reviewers the choice to opt into open identities review systems [22].

A very recent Elsevier survey of authors engaged in a pilot study of publishing review reports (with five journals involved and number of participants unknown) has found that of reviewers who accepted the invitation to review and publish reports, 76% said that open reports had no effect on the wording of their review. Of those who declined, the vast majority (91%) said their decision was not influenced by the open review. A third of editors involved in the pilot said they identified improvements in the overall quality of the review reports [27].

Finally, Nicholson and Alperin’s small survey (n = 79) of self-selected participants found that more than 75% of respondents believed that it would take no extra effort or only moderate extra effort to make their peer reviews suitable for public posting. Amongst their key motivations for doing so would be if tenure/promotion committees explicitly valued them and if their peers also published theirs [28].

## Materials and methods

### Survey instrument

The survey was conducted via an openly accessible online questionnaire (using the scientific survey platform SoSci, www.soscisurvey.de). Questions for the survey were developed through a review of the literature, discussion and feedback gained during the OpenAIRE Workshop “Open Peer Review—Models Benefits and Limitations” which took place in Göttingen, Germany on 7 June 2016 [29]. Additional input was crowdsourced via Twitter. The key survey foci were: attitudes to OPR, levels of experience with OPR, and definitions of OPR (including feedback on our OPR terminology/definition). A pilot version of the survey was created and disseminated to expert colleagues. Feedback and comments from six experts was then incorporated into a final revision of the survey.

The aim was to keep the average time to complete the survey to no more than 15 minutes in order to maximize full responses. The resulting survey instrument included a total of 18 questions. Questions 1–5 gathered demographic data. Questions 6–8 assessed overall levels of satisfaction with the peer review system used by scholarly journals and assessed general attitudes to open access, open data and OPR. Questions 9–15 then dug down into respondents’ levels of experience with and their opinions of each of the individual traits of OPR. Questions 16–17 sought feedback on a proposed definition of OPR. Finally, question 18 offered respondents the opportunity to leave free-text comments.

### Ethics

The survey instrument and plans for data collection/processing were approved by the Ethics Commission of the University of Goettingen (https://www.uni-goettingen.de/de/534983.html). All participants were informed through the survey website that the survey was anonymous and voluntary, that all data would be kept confidential and evaluated anonymously in compliance with the principles of the European Data Protection Directive, and that the purpose was to analyse the views of disciplinary communities regarding the provision/use of open peer review. Participants were informed that the study results and underlying data was to be published. To secure privacy all data was collected via a web survey (SoSci, www.soscisurvey.de) and analysed anonymously. In particular, aggregation was already built into the questionnaire (age groups, world regions, disciplinary areas, no gender category, no details about academic status, etc.) and no IP addresses were collected. All free-text data was aggregated and analysed separately.

### Processes and timeline

**Fig 1:**
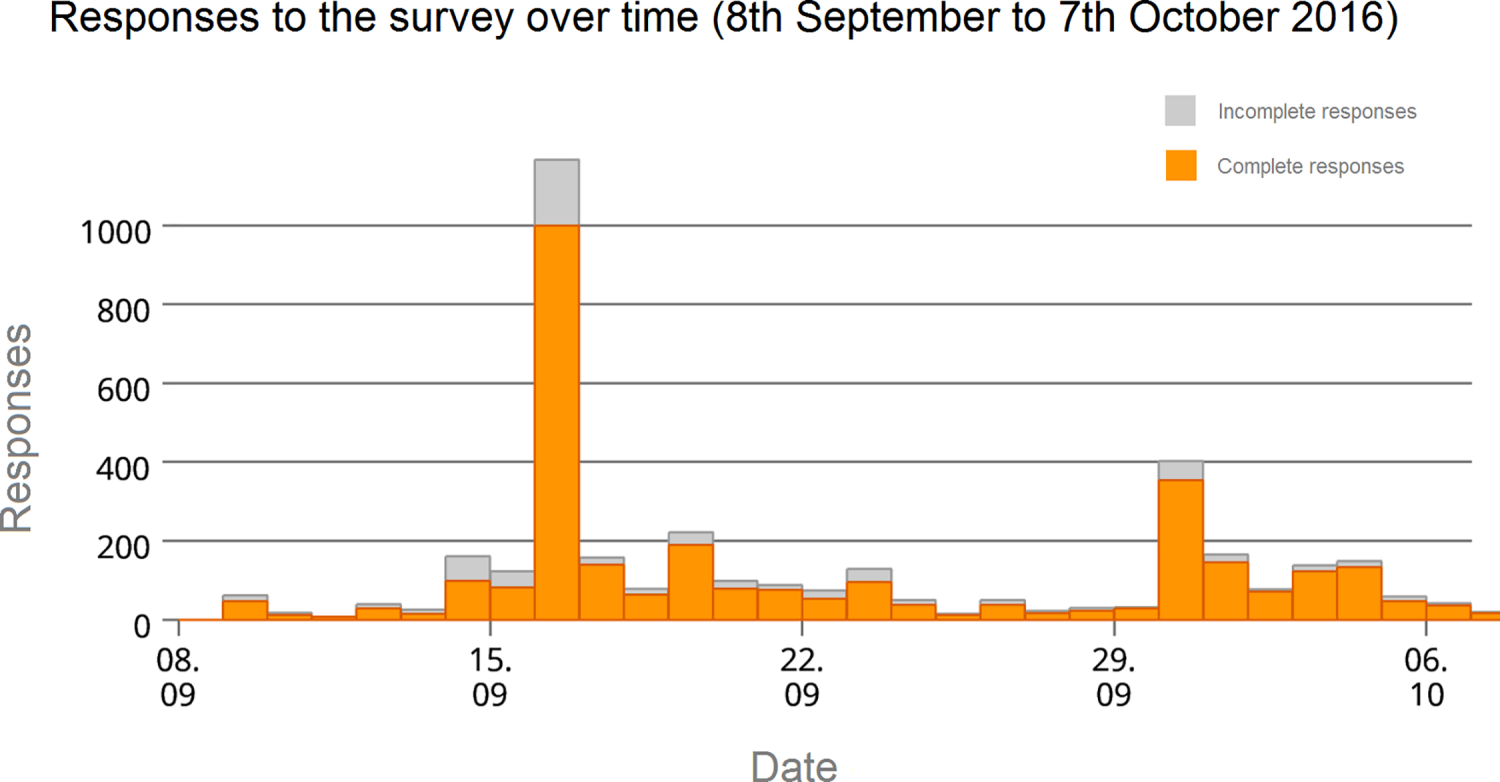
Responses to the survey over time (8th September to 7th October 2016).

The survey was open online from 8^th^ September to 7^th^ October 2016 and received a total of 3062 complete responses (a further 635 responses were discarded as incomplete). The survey was open to all wishing to take part and distributed via social media, scholarly communications mailing lists, publisher newsletters and, in one case, a publisher internal mailing list (Copernicus Publications). The latter, which directly targeted around 41000 authors, reviewers and editors, created a significant spike in responses (Fig 1), generating almost a third of the total responses in a single day. Overall, we consider 3062 responses for the analysis, constituting all complete survey responses.

### Study limitations

Given the mode of dissemination, it is likely that the sample is somewhat skewed towards those with more experience of and interest in open access publishing and new publishing models than would otherwise would have been the case in a randomized trial.

The study asked respondents to indicate whether they had experience of OPR as author, reviewer, editor or publisher, but did not explicitly specify which role they should assume when answering questions regarding their attitudes to OPR. As it may be expected that respondents’ considerations and hence responses might differ depending on their assumed role, this is acknowledged as a potential limitation of this study.

Further, it is important to remind ourselves that attitudes may not necessarily translate to practice—there may be large differences between what people say and what they actually do. Moreover, as a reviewer of this paper rightly pointed out, the views of participants should not be the sole steer of decision-making. It may be, for example, that distinct groups could be in favour of traits of OPR which would either conflict with each other or be somehow detrimental to the functioning of the larger system. Finally, some may simply be not well-informed about the issues. All of which is to say that our results should not be taken as a roadmap for OPR implementation, but rather as a further contribution to the evidence-base upon which policy decisions regarding (open) peer review should be taken.

## Results

### Demographics

**Fig 2:**
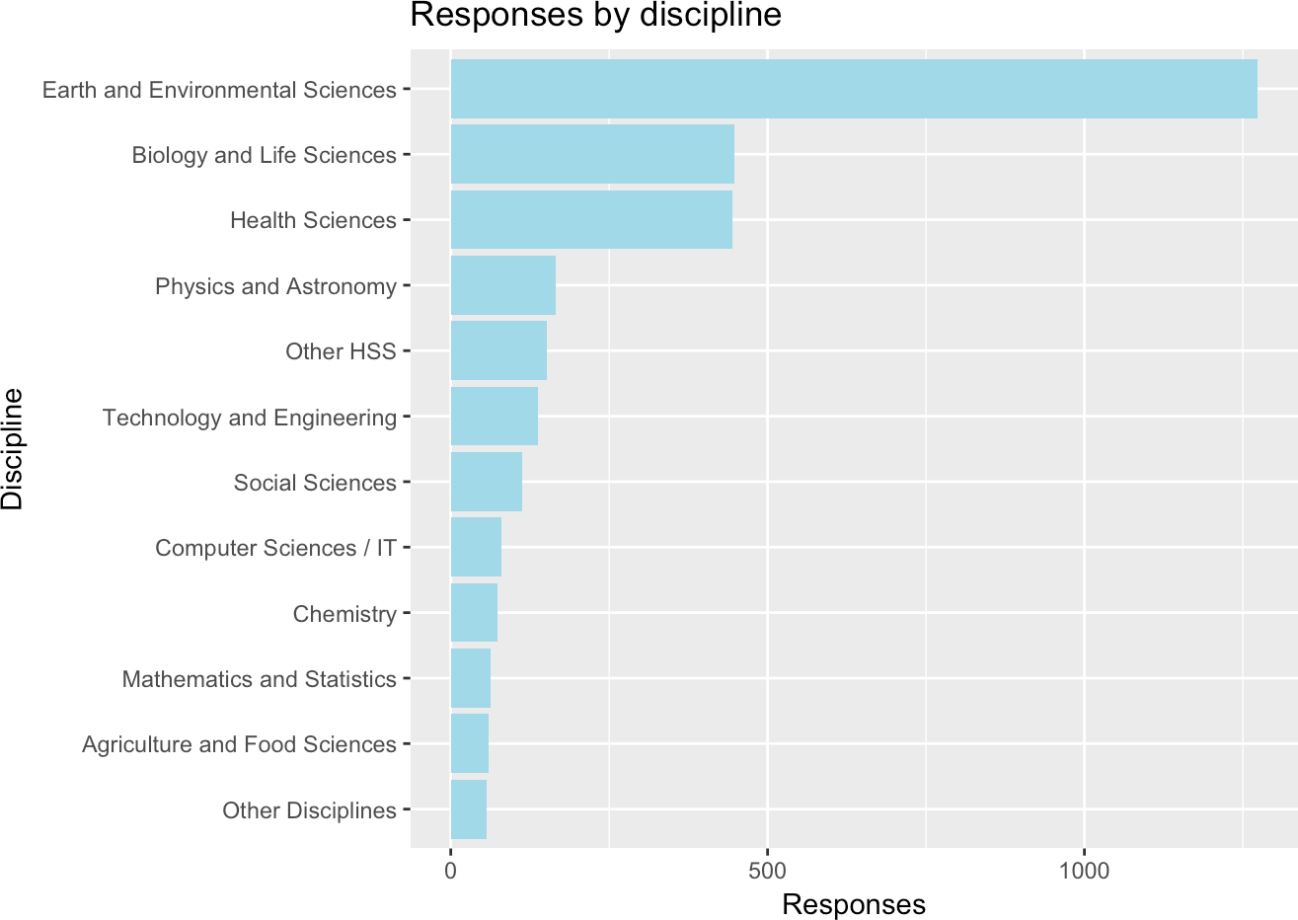
Responses by discipline (HSS disciplines clustered).

- **Age:** The majority of respondents provided information about their age group (n = 3049): 15 were under 24 (0.5%), 555 between 25 and 34 (18.2%), 918 between 35 and 44 (30.1%), 738 between 45 and 54 (24.2%), 573 between 55 and 64 (18.8%), and 250 over 65 years old (8.2%).
- **Region:** Respondents residing in Europe comprised 61.0% of total responses, followed by North and South America (22.4%), Asia (9.9%), Oceania (4.0%), and Africa (2.6%).
- **Discipline:** Responses by discipline were heavily skewed towards the natural sciences, which is due to the chosen mode of dissemination of the survey (cf. Table 1 and Fig 2). Overall, there were 89.6% (2743 responses) responses from science, technology and medical (STM) disciplines versus 8.6% (263 responses) responses from the social sciences and humanities (SSH), and 1.8% belonged to other disciplines (56 responses). When comparing STM and SSH responses we will leave out the cohort of other disciplines.

**Table 1:** Responses by discipline.

Discipline	Frequency	Percentage
Earth and Environmental Sciences	1274	41.61
Biology	447	14.6
Health Sciences	444	14.5
Physics	165	5.39
Technology and Engineering	137	4.47
Social Sciences	112	3.66
Computer Sciences / IT	80	2.61
Chemistry	73	2.38
Mathematics and Statistics	63	2.06
Agriculture and Food Sciences	60	1.96
Other disciplines	56	1.83
Psychology and Philosophy	50	1.63
Languages and Literature	35	1.14
History	23	0.75
Economics	18	0.59
Arts and Architecture	14	0.46
Law and Political Sciences	11	0.36

### Roles in scholarly communication and experience with OPR

Our respondents reported having strong experience of academic authorship and reviewing. Of all respondents, when asked about their roles in scholarly communication (multiple choices were allowed, on average about 2.4 options were selected) 2923 as author (95.5%), 2681 as reviewer (87.6%), 1326 had acted as editor (43.3%), 137 as publisher (4.5%), and 92 in other roles (3%).

**Fig 3:**
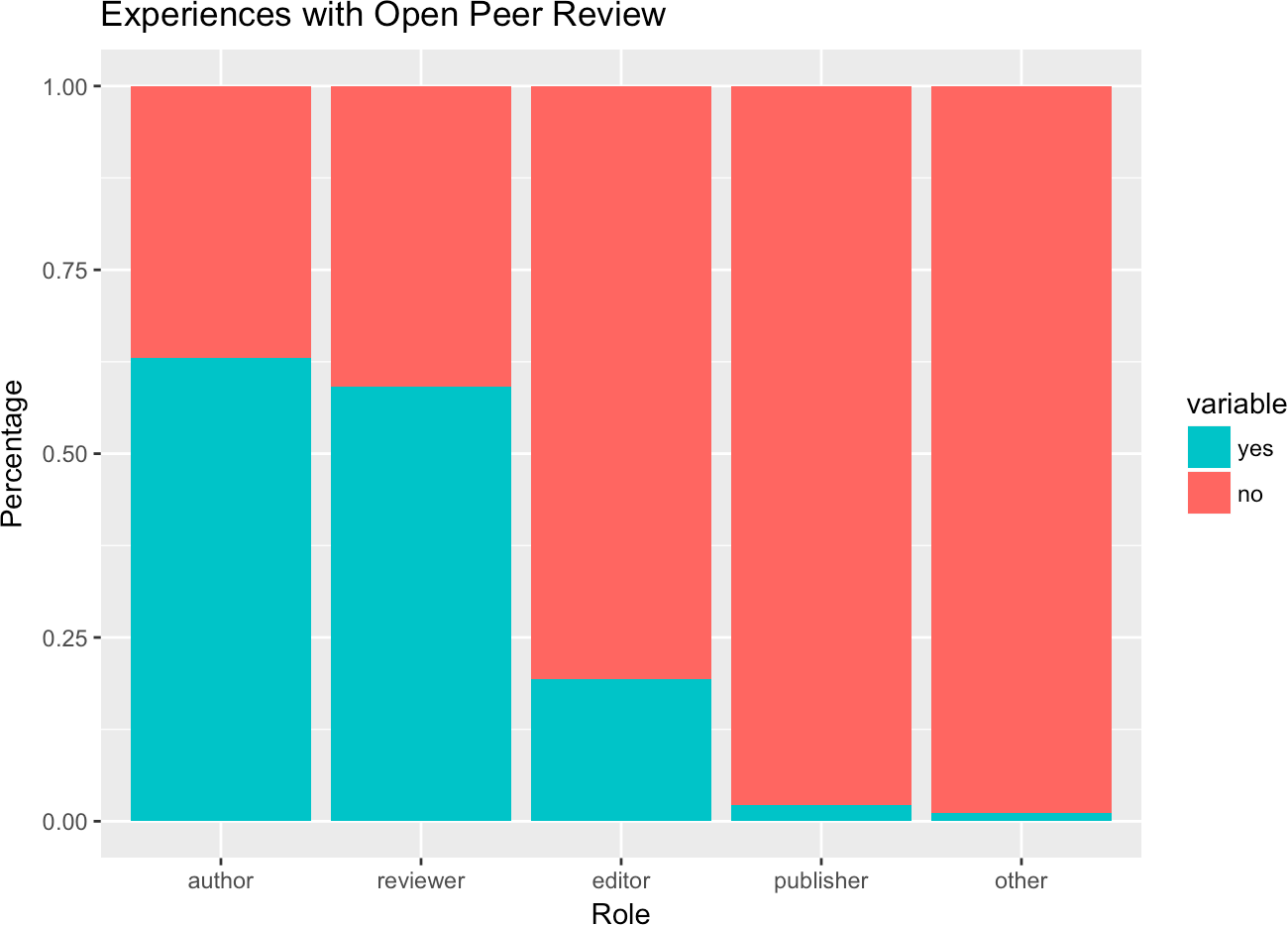
Experiences with OPR by role.

Of all respondents, when asked about their experience with OPR (multiple choices were allowed, on average about 1.9 options were selected) 594 had acted as editor (19.4%), 1930 as author (63%), 68 as publisher (2.2%), 1808 as reviewer (59%), and 35 in other roles (1.1%) (cf. Fig 3). Overall, over three out of four (76.2%, n = 2333) of all respondents reported having some experience with OPR as an editor, author or reviewer.

**Fig 4:**
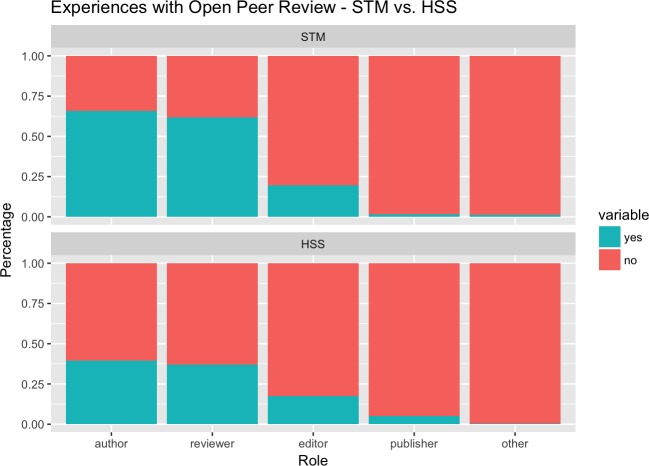
Experiences with OPR—science, technology and medicine (STM) vs. Humanities and Social Sciences (HSS).

Comparing respondents with STM vs. HSS backgrounds different levels of experience with OPR could be observed (cf. Fig 4). Almost 2/3 (1807 respondents, 65.9%) of all STM respondents had experience with OPR as an author, four out of five as a reviewer (1692 resp., 61.7%), about 1 out of five (540 resp., 19.7%) as an editor, and a few as a publisher (48 resp., 1.8%). In HSS subjects, levels of experience with OPR as authors and reviewers was substantially lower: about two out of five HSS respondents had OPR experience as an author (104 resp., 39.5%) and just over a third as a reviewer (97 resp., 36.9%). However, the share of respondents with OPR experience as an editor was at almost the same level (46 resp., 17.5%) and somewhat higher as a publisher (13 resp., 4.9%).

**Fig 5:**
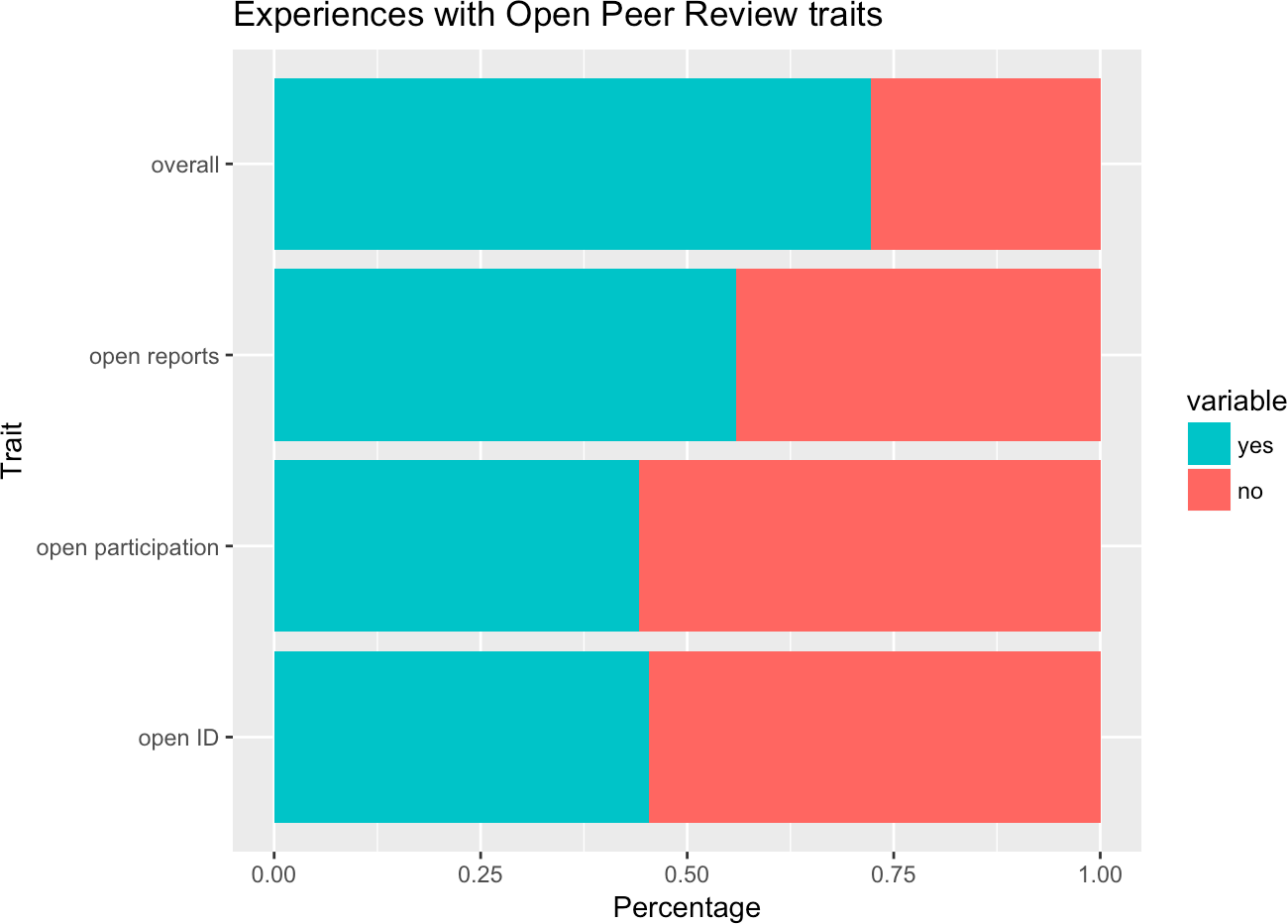
Levels of experience with OPR as author and/or reviewer.

Regarding particular OPR traits, a majority (55.9%) had had experience with open reports, but high numbers also reported experience with open identities (45.4%) and open participation (44.2%). Overall, 72.2% of all respondents (n = 3062) had contributed to one of these OPR activities (cf. Fig 5 and Table 2).

**Table 2:** Level of experience with OPR as author and/or reviewer.

	Overall	Open reports	Open identities	Open participation	Overall
**yes**	Freq	2211	1712	1389	1354
** **	Perc	72.2%	55.9%	45.4%	44.2%
**no**	Freq	851	1350	1673	1708
** **	Perc	27.8%	44.1%	54.6%	55.8%

### General attitudes to Open Science

**Fig 6:**
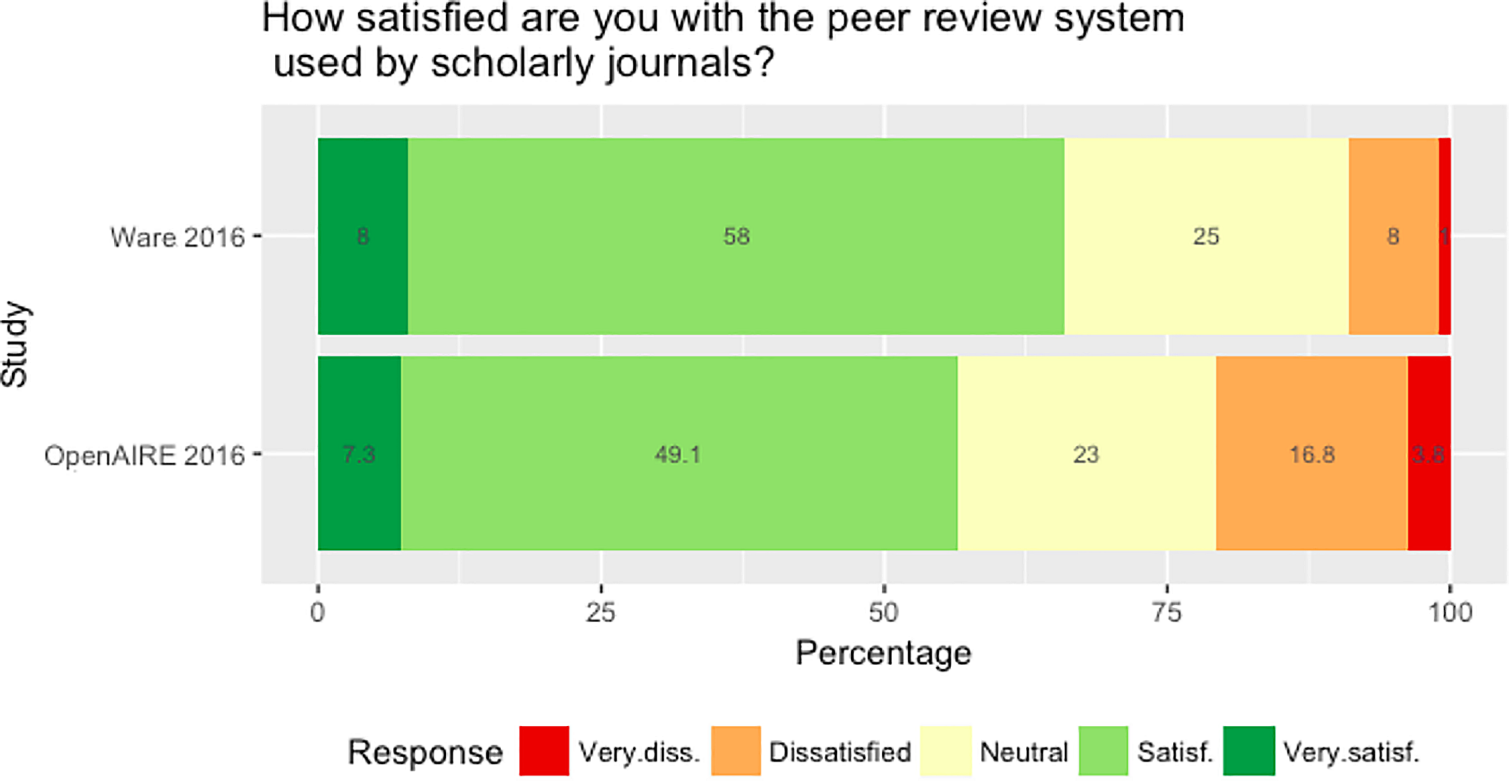
Overall satisfaction with peer review: Ware (2016, n = 2004) vs. OpenAIRE study (2016, n = 3001).

We began by asking respondents to state how satisfied they were with the current peer review system. Although there is no single monolithic peer review process, we here assume respondents understood this to mean “traditional” peer review (single/double blind, unpublished review reports, closed participation procedures, etc.). This question was chosen to replicate a question asked in previous studies [22, 26]. In part this was done to monitor if and to what extent our data collection method may have skewed our sample towards an audience more receptive to innovations in peer review. Although our respondents were generally in favour they were markedly less enthusiastic than those in previous studies, which found that between 65–69% of respondents were either satisfied or very satisfied with the current system, while just 9–12% reported being dissatisfied or very dissatisfied. This contrasts with our sample, where 56.4% reported being satisfied/very satisfied, while 20.6% were dissatisfied/very dissatisfied (cf. Fig 6 and Table 3).

**Table 3:** Overall satisfaction with peer review: Ware (2016, n = 2004) vs. OpenAIRE study (2016, n = 3001).

		Very satisfied	Satisfied	Neutral	Dissatisfied
**Ware 2015**	Freq	160	1162	501	160
** **	Perc	8.0	58.0	22.2	8.0
**OpenAIRE 2016**	Freq	218	1474	691	503
** **	Perc	7.3	49.1	23.0	16.8

When asked about the current state of scholarly communication there seems to be a reasonable level of satisfaction: of those who expressed an opinion almost every second respondent believed that scholarly communication works well (45.1%), while almost a third disagreed (31.6%).

**Fig 7:**
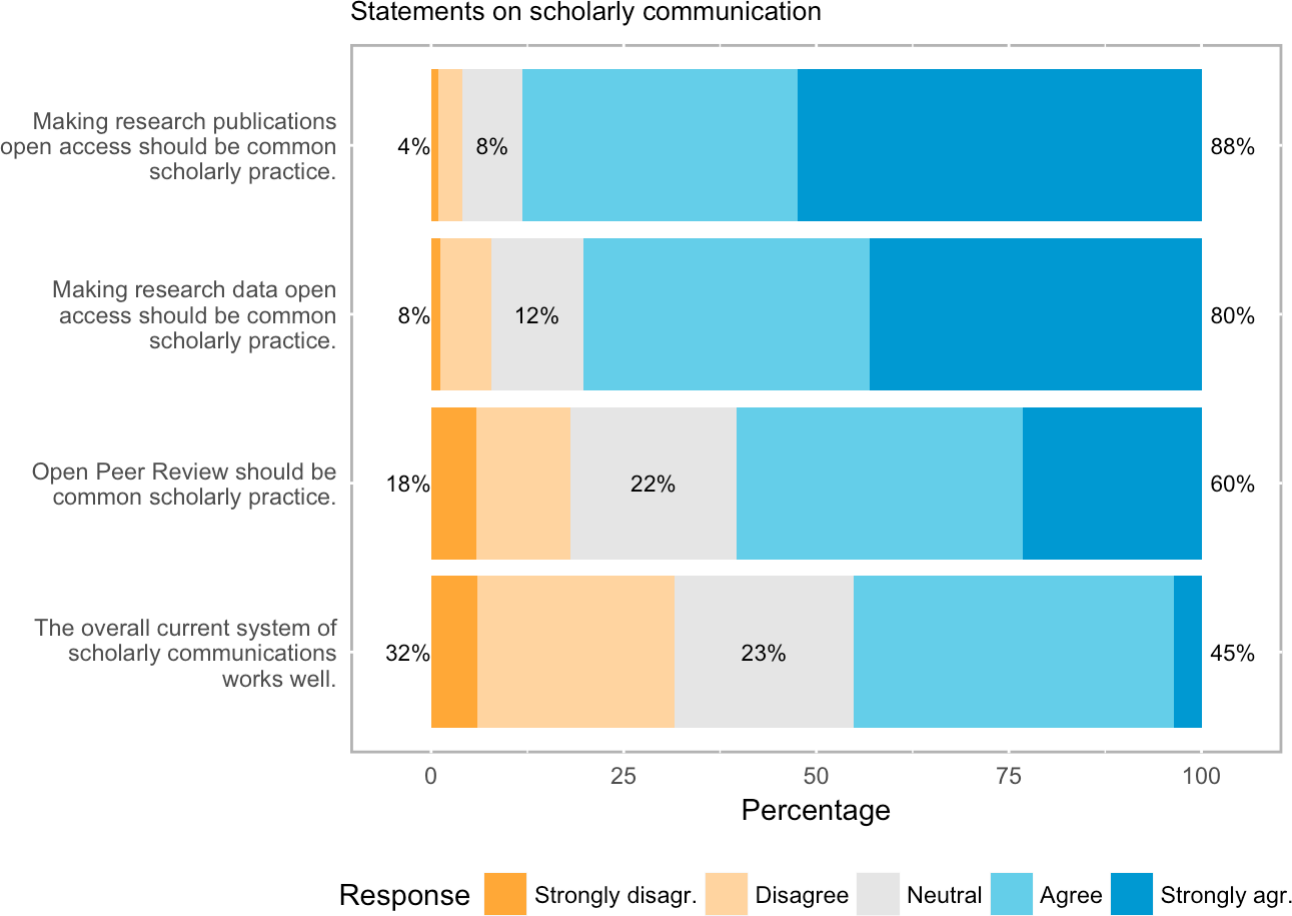
General attitudes towards aspects of open science.

Our sample was heavily in favour of Open Access to publications (88.2% agreed while only 4.1% disagreed) and research data (80.3% agreed, 7.8% disagreed), and a majority was in favour of OPR. Although enthusiasm was lower for the latter, still 60.3% (1733 respondents) believed that OPR should be common scholarly practice, while just 18.1% (518 respondents) disagreed. The questions on general attitudes to Open Science provoked decisive responses, with fewer than 50 “don’t know” responses for three out of the four questions; the “don’t knows” for the fourth question, on OPR, were higher, however (6.3%, 194 respondents, not considered for Fig 7).

We combine the negative (strongly disagree / disagree), neutral vs. positive (strongly agree / agree) attitudes for contrasting views on open access, open access to research data and OPR. Undecided sentiments, i.e. those who indicated “don’t know”, are not taken into account, however, for Open Access the share of these sentiments is very low with only 1.1% (34 respondents), while for open access to research data and OPR the share is quite substantial, with 10.1% (308 respondents) and 23% (703 respondents) of respondents respectively. The contingency tables indicate that these preferences may not be independent, positive attitudes often seem to come together: about four out of five (82.7%, n = 2264 out of 2739 which expressed an opinion) of all respondents support both open access to publications and research data, and a strong majority supported both open access and OPR (70.1%, n = 1647 of 2349 respondents), and similarly for open access to research data and OPR (70.2%, n = 1521 out of 2166 respondents) (cf. Tables 4, 5 and 6). Based on this observation we test the following hypothesis: Attitudes towards open access (OA) to publications and towards open access to research data are independent. We apply a Chi-square test of independence while noting that this test is highly sensitive to the sample size. For small samples it may fail to detect an association as significant and conversely for large sample sizes it may find statistical significance although the differences are small and uninteresting. In the cases under investigation there are large proportional differences across the categories (cf. Tables 4, 5 and 6). A Chi-square test of independence shows that the hypothesis stated above can be rejected (X-squared = 1459.3, df = 4, p<0.001). Similarly, the hypothesis that the variables of attitudes towards open access to publications and OPR are independent can be rejected (X-squared = 1236.2, df = 4, p<0.001), and finally also for open access to research data and OPR (X-squared = 773.81, df = 4, p<0.001). It must be noted that although the variables are related, causation in either direction cannot be inferred.

**Table 4:** Contingency table of attitudes to open access to publications vs. open access to research data.

OA to publ. / OA to data	negative	neutral	positive
**negative**	56	0	43
**neutral**	32	101	103
**positive**	140	266	2264

**Table 5:** Contingency table of attitudes to open access to publications vs. OPR.

OA to publ. / OPR	negative	neutral	positive
**negative**	73	0	24
**neutral**	70	108	58
**positive**	368	0	1647

**Table 6:** Contingency table of attitudes to open access to research data vs. OPR.

OA to data / OPR	negative	neutral	positive
**negative**	111	0	62
**neutral**	28	55	18
**positive**	318	40	1521

**Fig 8:**
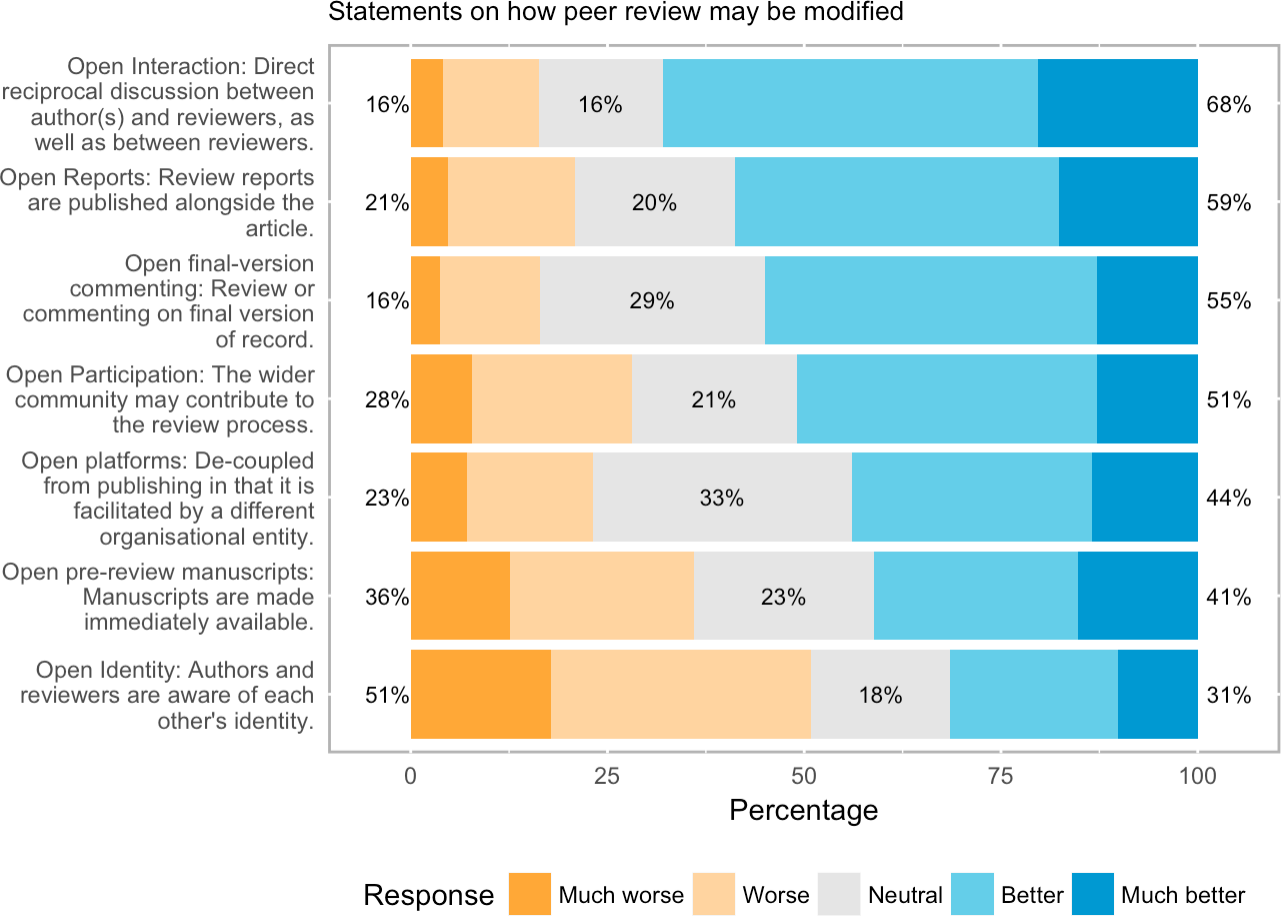
“Will”X”make peer review better, worse, or have no effect?”.

We next asked about attitudes to particular aspects of OPR (see Introduction above for description of the meaning of each of these traits as they were presented to respondents). Our respondents on the whole tended to think that most aspects would improve peer review (Fig 8). The clear exception is open identities, with 50.8% (1461 of 2878 respondents who expressed an opinion) of our respondents believing that open identities will make peer review worse or much worse. Given that open identities is the trait most commonly found in definitions of OPR (present in 110 of 122 definitions we studied), this is somewhat surprising and, from analysis of the free-text comments left by respondents, seems to reflect persistent concerns that opening reviewer identities will lead to a lack of control. In particular, protection from undue influence, making it more difficult for particularly junior researchers to give candid feedback for fear of possible reprisals from aggrieved authors.

Also of interest is the observed level of enthusiasm for a secondary aspect of OPR, namely open interaction where direct reciprocal discussion between author(s) and reviewers, and/or between reviewers, is allowed and encouraged. This aspect is supported by over two thirds of all respondents (68.0%, 1976 of 2904 expressing an opinion). Given that open interaction is a fairly incremental change compared to other more radical elements of OPR, this level of support suggests that this is presently the easiest route to “open” peer review processes.

Further aspects which were supported by about every second respondent are the publication of peer review reports (58.8%, 1722 of 2928 respondents), the ability to comment on the final version of record (55%, 1471 of 2676 respondents) and the wider participation of the community in peer review processes (51%, 1463 of 2870 respondents).

It must be noted that for some of these aspects the level of indecision was rather high, in particular for open platforms, where about a quarter of respondents opted for “don’t know” (cf. Table 7).

**Table 7:** Responses to statements on how peer review might be modified (sdv = standard deviation).

	mean, sdv		much worse	worse	neutral	better	much better	don’t know
**Open reports**	3.51	Freq	139	469	598	1201	521	134
** **	1.10	Perc	4.5	15.3	19.5	39.2	17.0	4.4
**Open identity**	2.73	Freq	512	949	508	615	294	184
** **	1.26	Perc	16.7	31.0	16.6	20.1	9.6	6.0
**Open participation**	2.28	Freq	222	582	603	1094	369	192
** **	1.15	Perc	7.3	19.0	19.7	35.7	12.1	6.3
**Open interaction**	3.68	Freq	119	353	456	1385	591	158
** **	1.06	Perc	3.9	11.5	14.9	45.2	19.3	5.2
**Open pre-review manuscripts**	3.08	Freq	354	655	640	729	430	254
** **	1.27	Perc	11.6	21.4	20.9	23.8	14.0	8.3
**Open final-version commenting**	3.48	Freq	98	342	765	1127	344	386
** **	0.99	Perc	3.2	11.2	25.0	36.8	11.2	12.6
**Open platforms**	3.27	Freq	157	354	729	677	298	847
** **	1.10	Perc	5.1	11.6	23.8	22.1	9.7	27.7

### Attitudes to open identities

**Fig 9:**
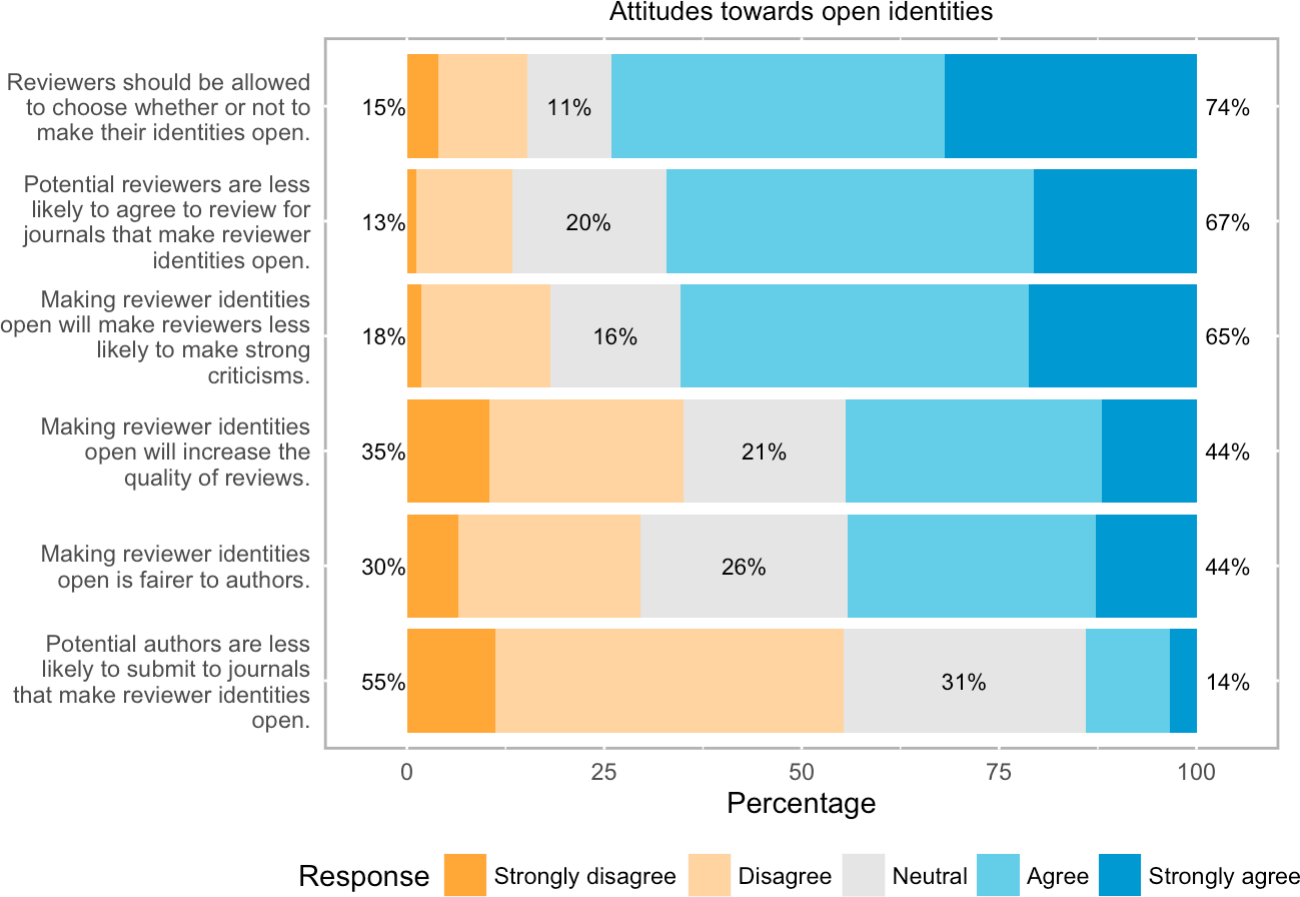
Attitudes towards open identities.

On the whole, the pushback against open identities that we observed above continued throughout respondents’ answers to questions on this theme (Fig 9). They strongly believed (73.9% agree/strongly agree, 2175 of 2944 respondents) that reviewers should be allowed to choose whether or not to make their identities open and that potential reviewers would be less likely to review for journals that practiced such peer review (67.2% agree/strongly agree, 1858 of 2767 respondents). Almost two thirds also believed that open identities would lead to reviewers being less likely to deliver strong criticisms (65.2% agree/strongly agree, 1909 of 2926 respondents). When comparing the subsets of those respondents with vs. those without OPR experiences (in any of the roles author, reviewer, editor or publisher) it turns out that most attitudes differ only marginally. However, when it comes to allowing reviewers to choose whether to reveal their identities support for this option is somewhat stronger for those *with* OPR experiences than for those without OPR experiences (73% agree/strongly agree vs. 64.5% agree/strongly agree).

However, our respondents did, on the whole, seem to believe that making reviewer identities open would increase review quality (44% agree/strongly agree vs. 35% disagree/strongly disagree, n = 2908). Moreover, roughly the same numbers believed that open reviewer identities is fairer to authors (44% agree/strongly agree vs. 30% disagree/strongly disagree, n = 2915). This indicates that although our respondents see worth in open identities peer review, they remain skeptical about its effects and advocate choice in its implementation.

Not surprisingly the statement that making reviewer identities open would make authors less likely to submit manuscripts was rejected by the majority of respondents, although almost a third indicated they were undecided on this issue.

### Attitudes to open reports

**Fig 10:**
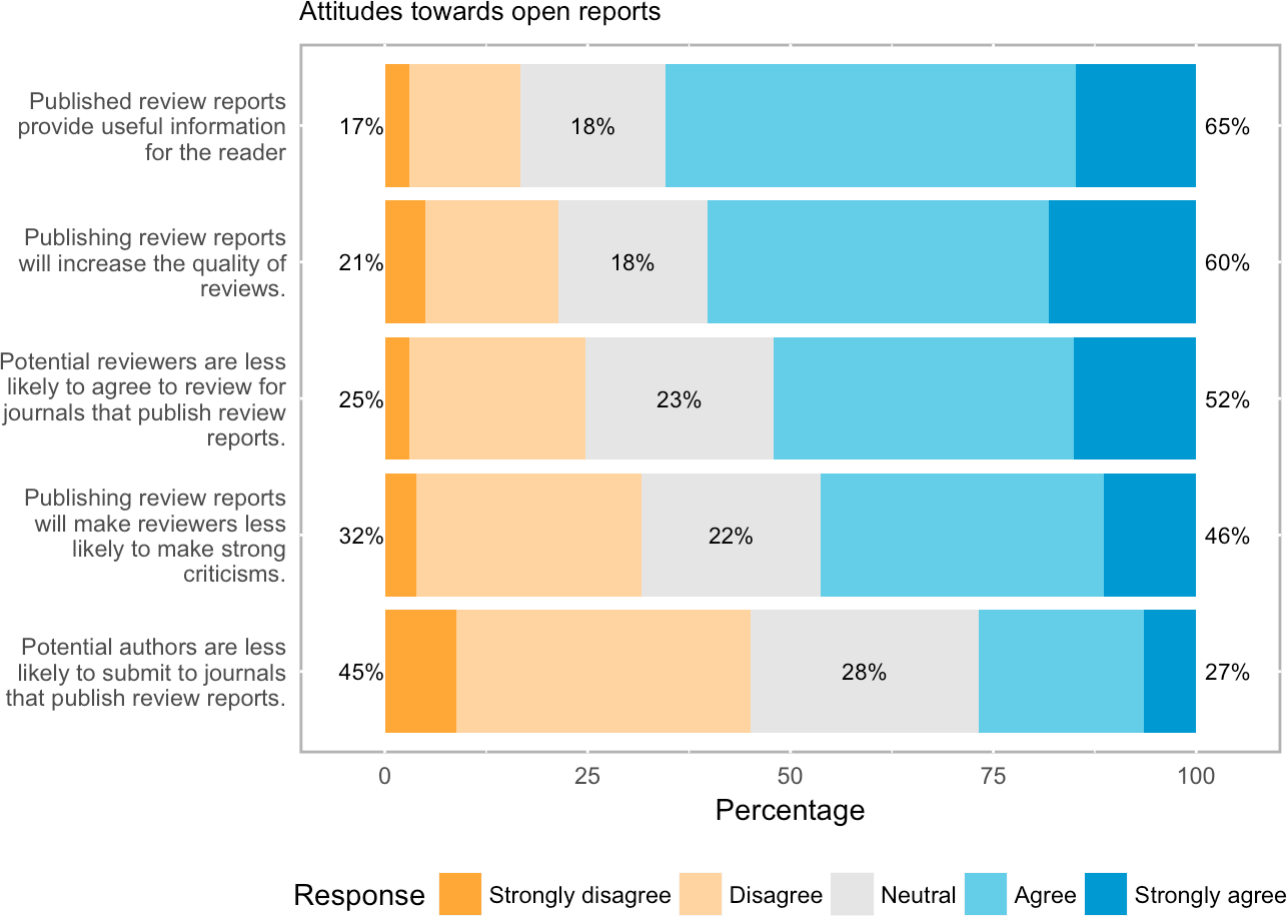
Attitudes to open reports.

Respondents generally see worth in publishing review reports (Fig 10). Almost 2 out of 3 respondents (65.4%, 1933 out of 2956 respondents) agree or strongly agree that they provide useful information for the reader, and 3 out of 5 respondents (60.2%, 1765 out of 2930 respondents) believe publishing reports will increase review quality. Respondents largely rejected the idea that open reports would be a disincentive for authors (over 1 out of 4 respondents agree or strongly agree). However, similar to open identities (although with a less pronounced effect), they believe that publishing reviews might disinhibit reviewers and lead to less strong criticisms.

### Attitudes to open participation

**Fig 11:**
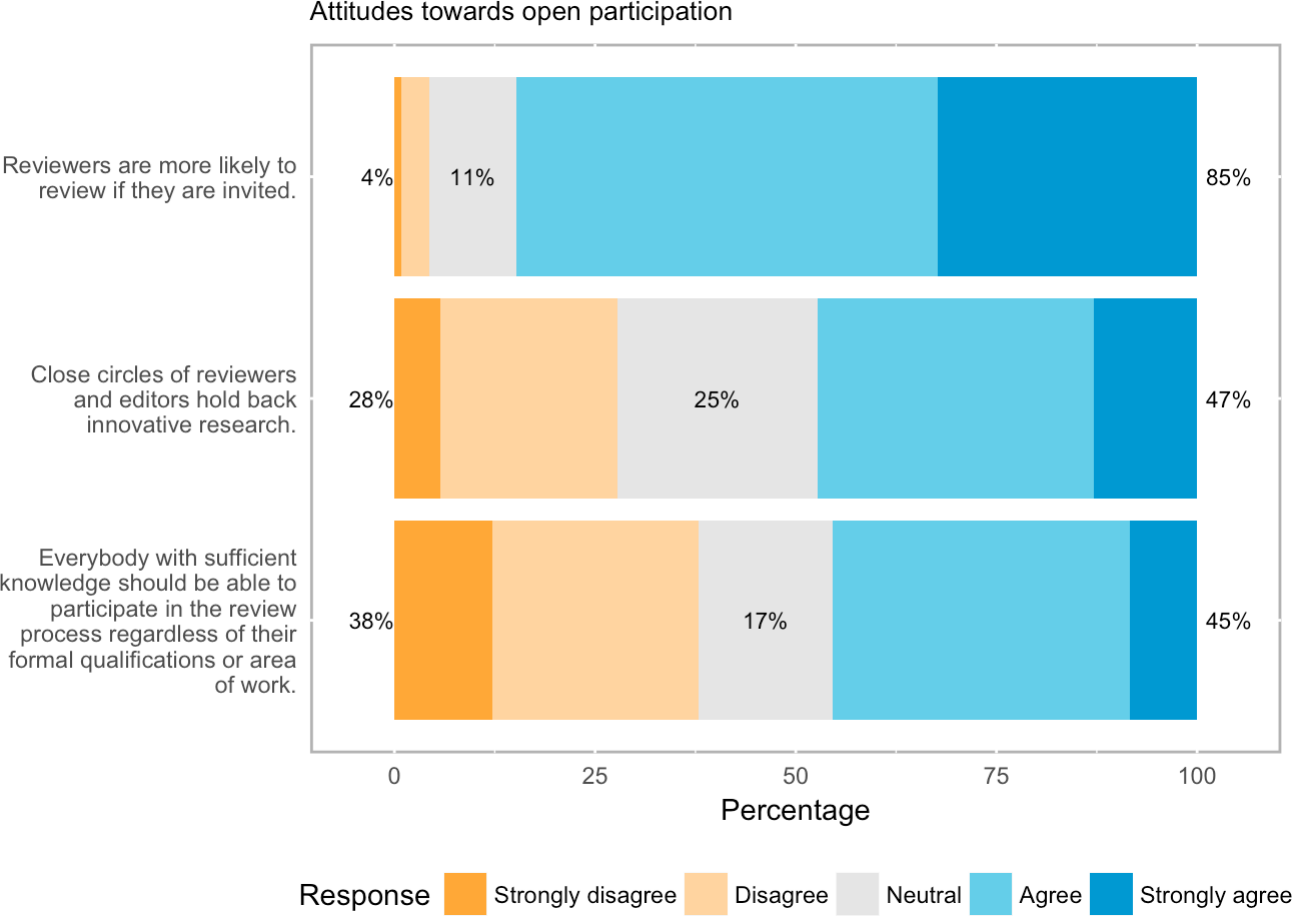
Attitudes to open participation.

Attitudes to open participation were mixed (Fig 11). Although only 27.7% (772 out of 2789 respondents) disagreed that close circles of reviewers hold back innovative research, respondents were not wholly enthusiastic about opening participation. There was a fairly even split to the suggestion that everybody, regardless of qualifications or background, should be able to participate in the review process (45% agree/strongly agree vs. 38% disagree/strongly disagree, n = 2913 responses). The recurrent concern about whether people will voluntarily participate was also present for our respondents, 85% (2460 out of 2902 respondents) of whom thought that reviewers are more likely to review if invited.

### Attitudes to other OPR traits

**Fig 12:**
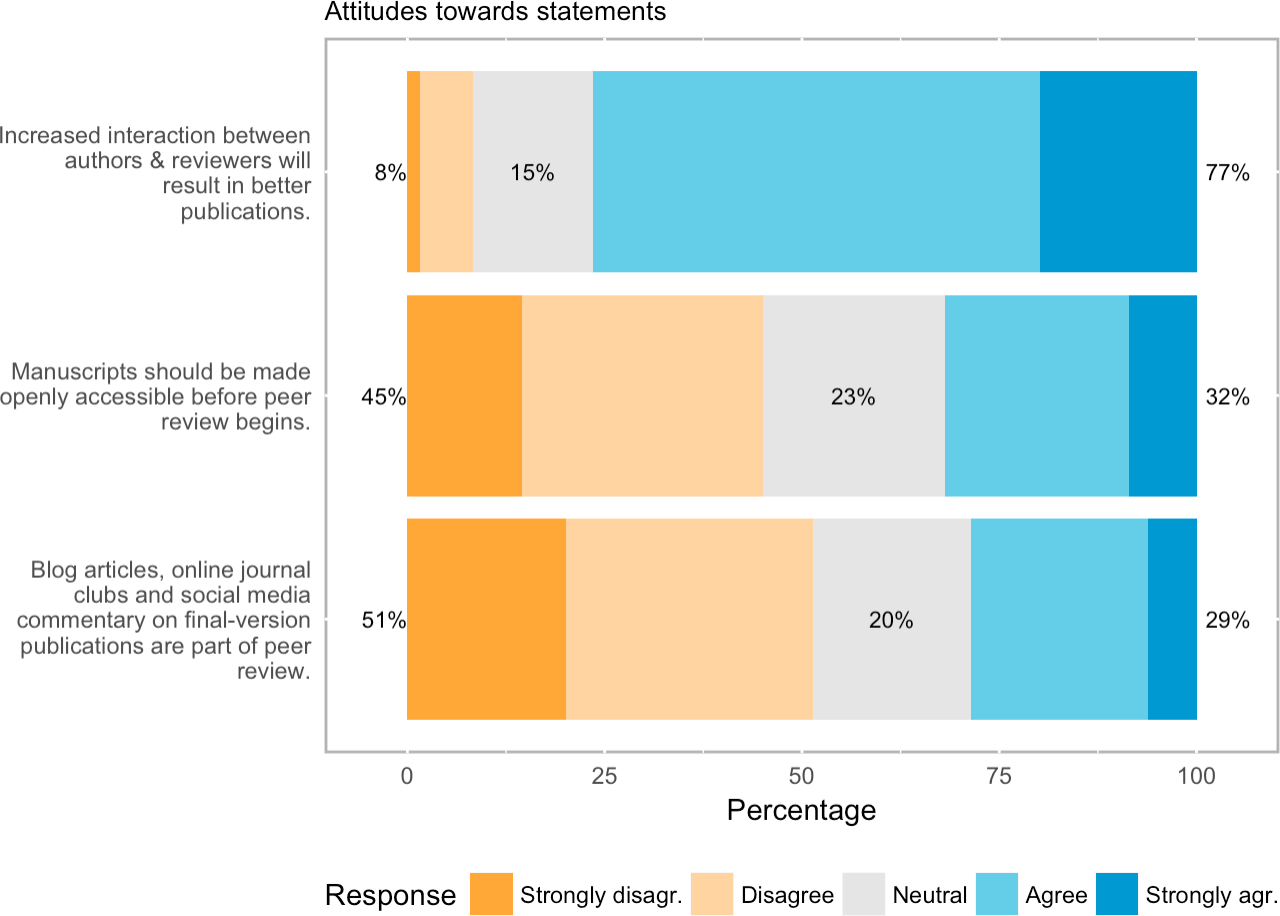
Attitudes towards other OPR traits.

A more positive attitude towards open interaction was reflected in the belief of three out of four respondents (76.5% agree/strongly agree, n = 2257 responses) that increased interaction between authors and reviewers will result in better publications (Fig 12).

Regarding making peer review processes more open in terms of time (open pre-review manuscripts or final version commenting), support was less pronounced. Modest support was observed for making manuscripts openly available before peer review begins (32%, 919 responses). Just over a quarter of respondents, meanwhile, believe that post-publication commentary (online commentary such as blog articles, journal clubs and social media commentary) forms a legitimate part of peer review (28.8%, n = 807 responses).

### Some disciplinary differences

**Fig 13:**
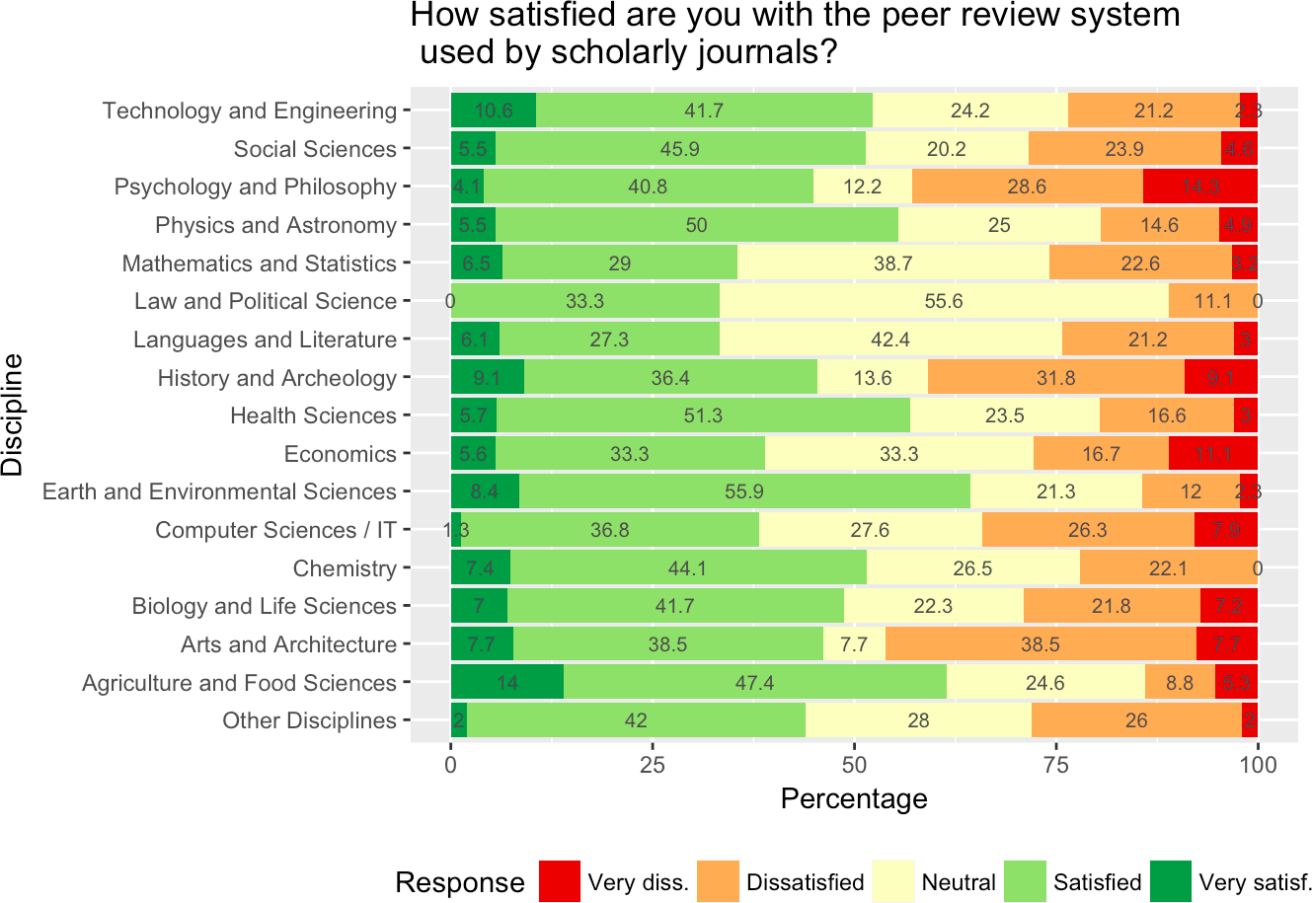
Degree of satisfaction with the peer review system across scientific disciplines.

Overall satisfaction with the peer review system used by scholarly journals seems to strongly vary across disciplines (Fig 13). Some disciplines seem to be rather satisfied while for some a high degree of dissatisfaction was expressed. However, it is possible that there was some self-selection, with those more critical of the current system choosing to participate in the survey. In addition, for some disciplines there were only few responses, e.g. in several humanities research areas (see Table 1). For the STM research areas a majority of respondents seem to be satisfied, e.g. respondents from the earth and environmental sciences (64.3% very satisfied / satisfied vs. 14.3% very dissatisfied / dissatisfied), agriculture and food science (61.4% vs. 14.1%), physics and astronomy (55.5% vs. 19.5%) and health sciences (57% vs. 19.6%). Exceptions were mathematics and statistics where only about one third of respondents (35.5%) seem to be satisfied while over one fourth (25.8%) are not satisfied, and similarly computer sciences / IT with only every third respondent satisfied, with a similar amount not satisfied (38.1% vs. 34.2% respectively). High levels of dissatisfaction were expressed by respondents from arts and architecture, history and archeology and psychology and philosophy (between 40 and 45% very dissatisfied / dissatisfied).

**Fig 14:**
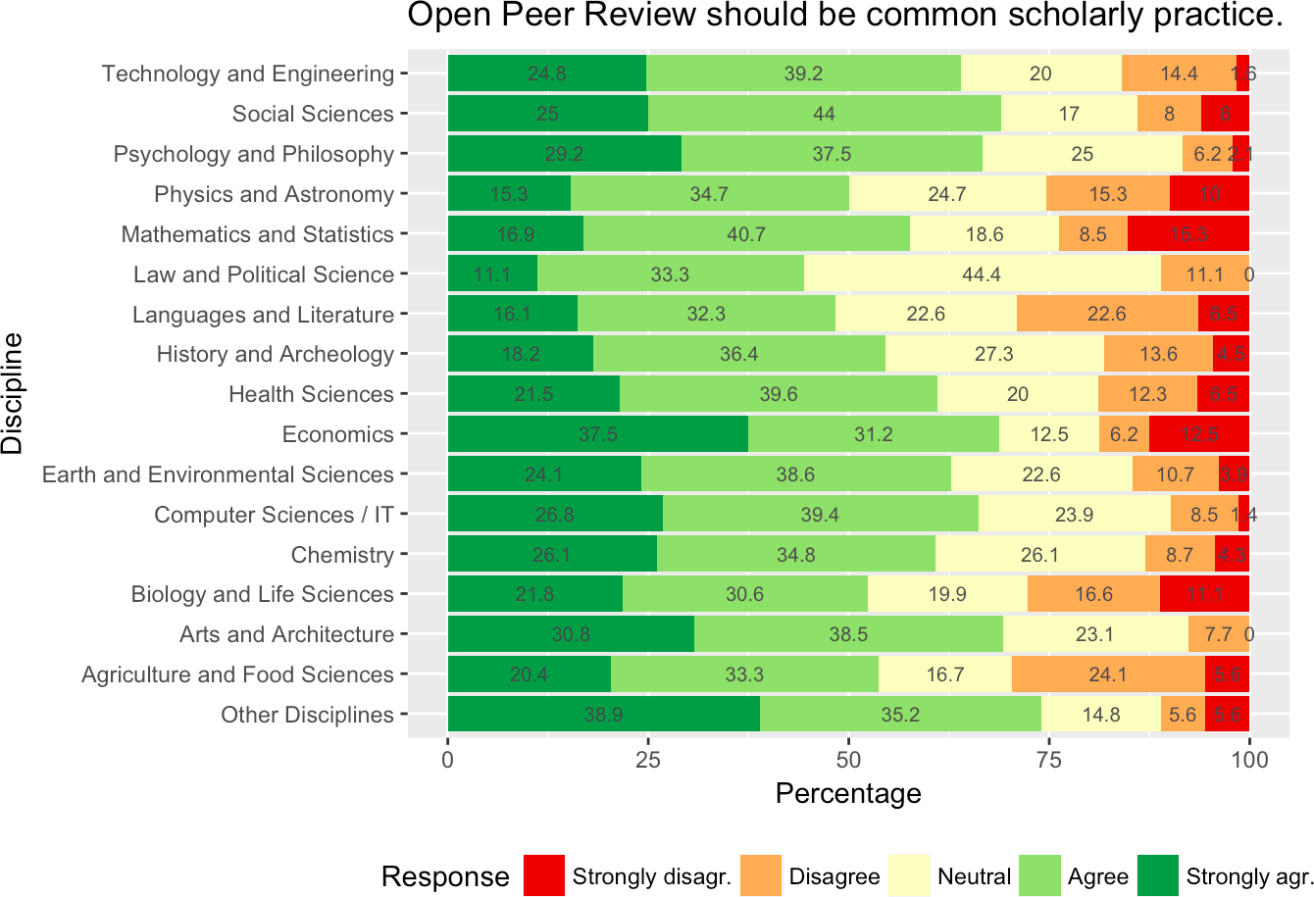
Views on OPR by scientific discipline.

When asked if OPR should be a common scholarly practice for most research areas a majority of respondents agreed or strongly agreed (Fig 14), although support for open access to publications and research data was stronger. Support was particularly high from economics, social sciences, psychology and philosophy, arts and architecture and other disciplines with over 2/3 of all respondents. The strongest reservations towards opening up peer review could be observed from agriculture and food sciences, languages and literature, biology and life sciences, physics and astronomy as well as mathematics and statistics with about one out of four respondents disagreeing or strongly disagreeing.

**Fig 15:**
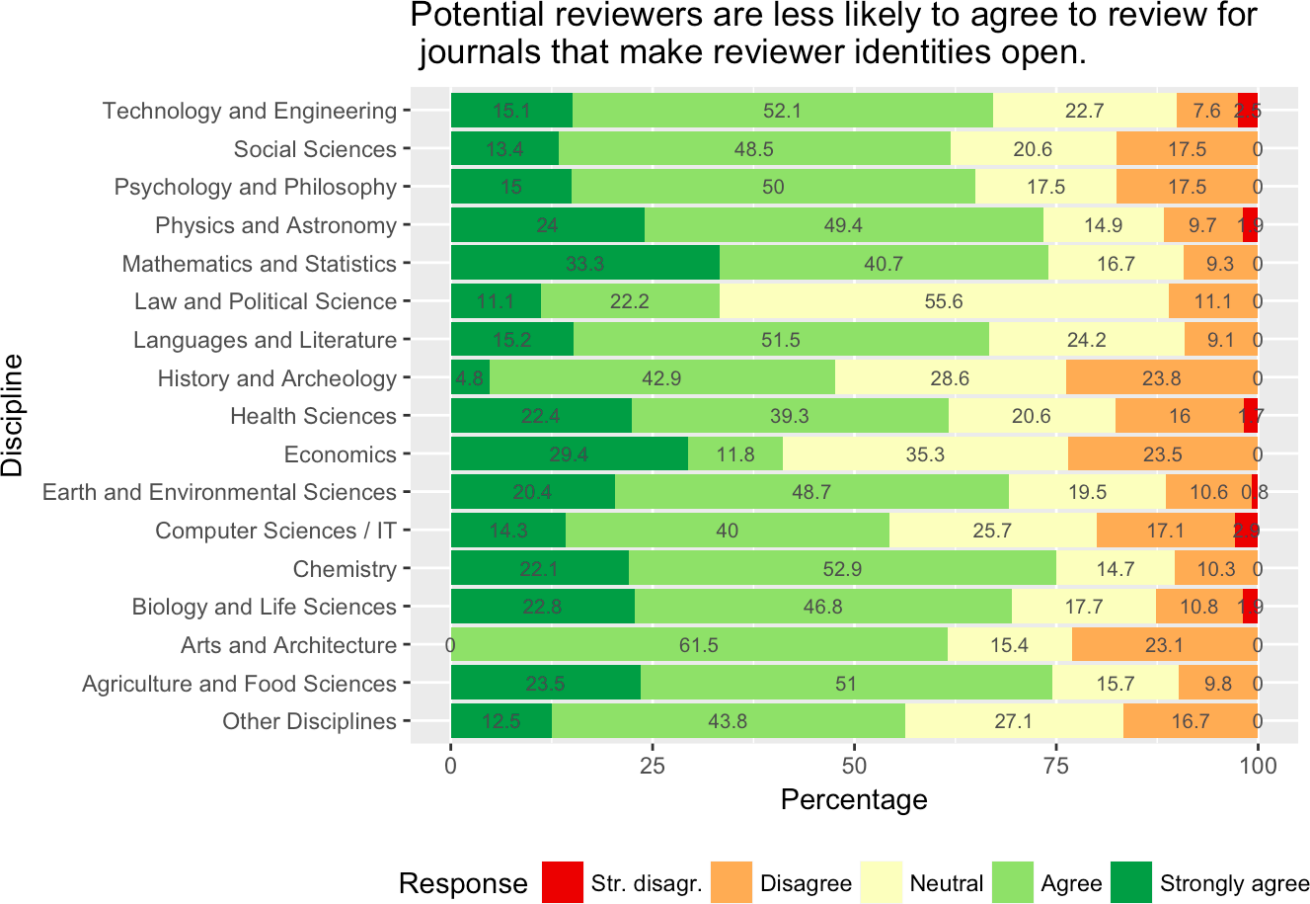
Responses by discipline regarding perceptions of reviewer’s willingness to review for journals which make reviewer identities open.

For almost all research areas a majority of respondents felt that making reviewer identities open will make reviewers less willing to review for such journals (between about every second and over 3 out of 4 respondents, cf. Fig 15). In a very few disciplinary areas, this perception was a little less pronounced: e.g., for the law and political sciences about every third respondent agreed or strongly agreed with this view.

### Generational differences

When asked if the current system of scholarly communication works well, there was significantly less agreement in the younger generation of researchers, in particular when comparing the under 35-year-olds with the generations 45 and older. For a MANOVA significance analysis we clustered the under 25-year-olds with the generation 25–34 as there were only very few responses for the younger cohort. Responses were ordinalised on a five-point scale from disagreement via neutral to agreement (1 = “strongly disagree”, 2 = “disagree”, 3 = “neither agree nor disagree”, 4 = “agree”, 5 = “strongly agree”).

Overall, the younger generations provided somewhat stronger support for the general statements on openness practices. There was a significantly stronger support for open access amongst those aged 35 and under compared with the generations 45–54 (p<0.001), 55–54 (p<0.001) and 65 and over (p<0.001). Also, the generation 35–44 expressed somewhat stronger support for open access than the generations 45–54 (p = 0.011) and 55–64 (p<0.001).

**Fig 16:**
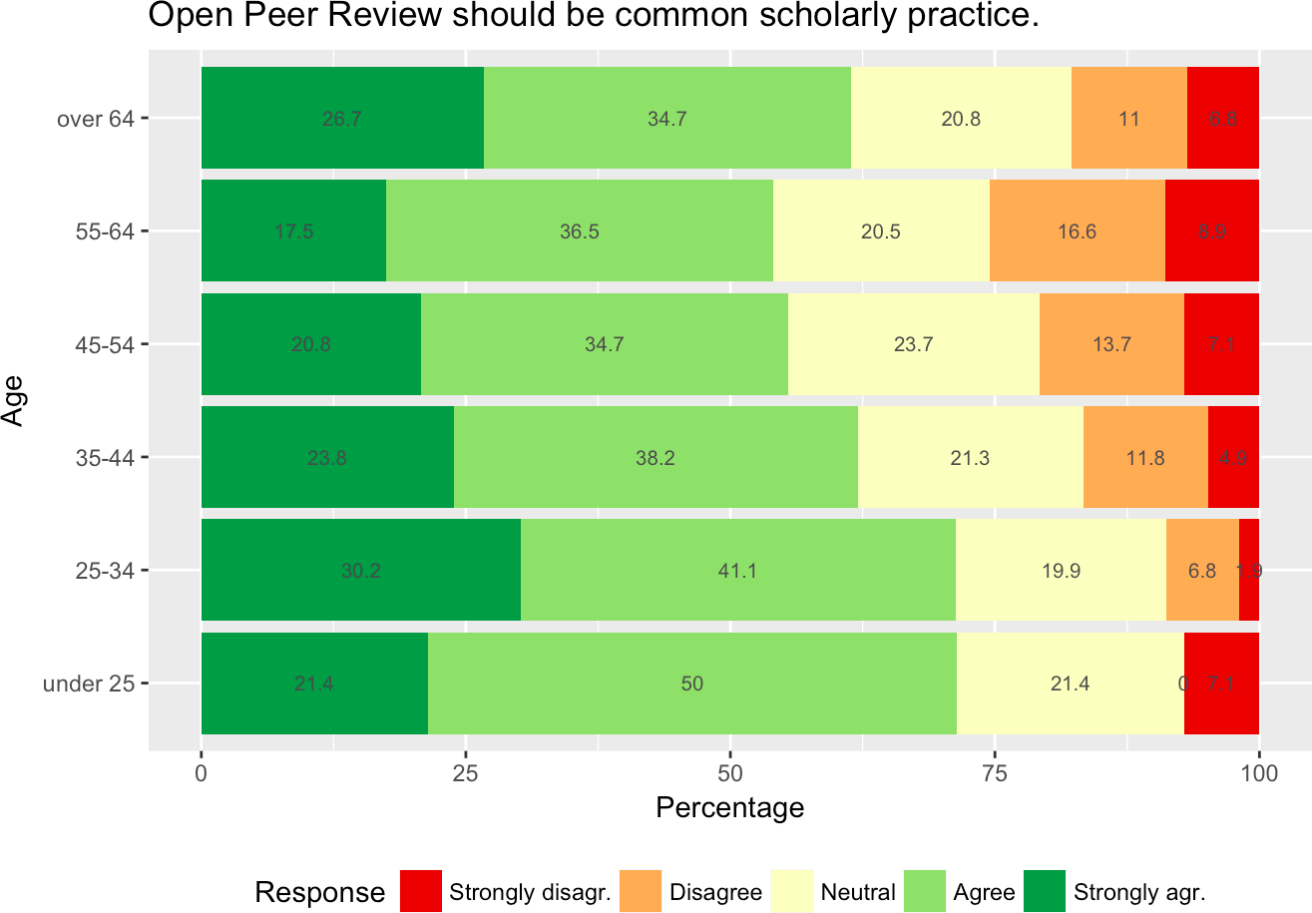
Views by age group on whether OPR should be common scholarly practice.

Making research data open access was significantly more strongly endorsed as a common scholarly practice by the under 35s compared to the generations 45 and older (p<0.05 in all three cases) and the 35–44 year-olds compared to the 55–64 year-olds (p<0.01). In addition, OPR was significantly stronger supported as a common scholarly practice when comparing the under 35 with all other age groups (p<0.05 in all cases) and when comparing the 35–44 and 45–54 and 55–64 year-olds (p<0.05 in both cases). For an overview see also Fig 16.

### Free-text comments

The last question of the survey invited participants to provide additional views on (open) peer review but also general comments on the design of the survey. In total, 901 of 3062 respondents entered something in this field. Removing 167 completed fields where content was entered to indicate no response (i.e., respondents who entered “no”, “nope”, “none”, “not”, “nothing”, “nil”, “not at this point”, “no other comments”, “N/A”, “.” or “-”) left 734 comments from respondents.

**Fig 17:**
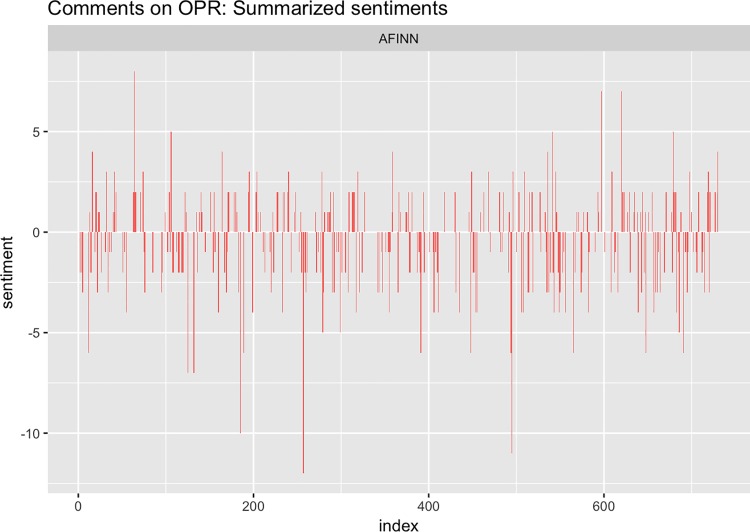
Comments on OPR–summarized sentiments.

A detailed analysis of these:

We assess these responses by summarising the general sentiment which was expressed by applying text and data mining methods [30]. We consider all bigrams based on the free text comments. Based on the AFINN sentiment lexicon (created and maintained by Finn Årup Nielsen, available via R’s tidytext library [31]) we assign sentiment values to the second word of all bigrams. As the word “blind” is coded as negative in the AFINN lexicon we leave out combinations in which “blind” is the second word. Moreover, we change the direction of the sentiment if the first word indicates a negation (“no”, “not”). These values are then summarised to a sentiment score per response (Fig 17). Overall, slightly more negative (52.7%, 166 out of 315 responses) than positive comments (47.3%, 149 responses) were provided. In addition, there were more than twice as many strongly negative ones than strongly positive ones (16 vs. 6 comments with score less than or equal to -5 or larger or equal than 5).The authors plan to undertake further analysis of these comments in a future, qualitative study of OPR.

## Discussion and future developments

Our results suggest that OPR is currently moving to the mainstream, with high levels of experience amongst authors, reviewers and editors, most of whom have positive attitudes toward it. A clear majority (60.8%) of respondents believe that OPR should be common scholarly practice, although support for the various traits varied and there was a negative view of the core trait of open identities. Support for OPR is not as strong as for other areas of Open Science, however. Almost 9 in 10 of our respondents thought Open Access to publications should be common scholarly practice, while four fifths of respondents thought the same of open research data. Nonetheless, our findings reveal that being in favour of OPR is associated with being in favour of Open Access and Open Data, suggesting that researchers are increasingly in favour of the Open Science agenda, disclosing their identities). Hence, it might be postulated that as knowledge about and experience with OPR grows, it will come to have similar support as these more established elements of Open Science. Certainly, there is a generational element, with significantly stronger levels of support for OPR amongst younger generations.

Our sample demonstrated a very high level of familiarity with OPR—more than three out of four respondents reported having some experience with OPR as an editor, author or reviewer (19% as editors, 63% as authors and 59% as reviewers). As might be expected of what are (in relation to scholarly communication) often perceived to be more conservative disciplines, levels of experience with OPR as authors and reviewers was substantially lower amongst those from Humanities and Social Sciences. Regarding particular OPR traits, a majority (55.8%) had experience with open reports, but high numbers also reported experience with open identities (45.4%) and open participation (44.2%). While admittedly our open method of distribution likely skews towards those with more experience of and interest in open access publishing and new publishing models than would have been the case in a randomized trial, and hence these levels of experience can be assumed to be somewhat higher than in the general population of researchers, these are still impressive results which demonstrate emphatically that OPR is no longer a fringe phenomenon. Moreover, the fact that our sample was perhaps more experienced with OPR than the average population lends extra credence to respondents’ reported views of their OPR attitudes, which are then more likely to be based on *a posteriori* experience than *a priori* supposition.

Respondents’ general enthusiasm for OPR was not uniform to all the traits of OPR, however. Asked to rate whether individual traits would make peer review better or worse, six traits found a majority of respondents in favour, with open interaction (68% in favour) the most popular. The least popular trait amongst our sample by a wide margin, and the only one which the majority believe will actually make peer review worse, was open identities (51% against, 31% in favour). In the remainder of this discussion we will address attitudes to each of these traits in turn.

### Open identities

Perhaps the most surprising finding in the survey was respondents’ negative reaction towards open identities peer review, where authors and reviewers are aware of each other’s identities. As previously stated, this was by far the most negatively perceived OPR trait, with a majority believing it would make peer review worse. This is especially surprising since open identities is often the hallmark trait of OPR within existing definitions within the literature [16]. As one of the reviewers for this paper points out, given the relatively high levels of personal experience with OPR reported by our cohort, this opposition is particularly striking and is to be taken as a point to be seriously addressed by those contemplating implementing open identity peer review systems. This pushback continued throughout respondents’ answers to questions on this theme, with strong beliefs that reviewers should be allowed to choose whether or not to make their identities open (74%) and that potential reviewers would be less likely to review for journals that practiced such peer review (67%). This was so despite seeming recognition of the potential benefits of open identities: asked whether making reviewer identities open would increase review quality, 44% agreed whereas 35% disagreed, and asked whether open reviewer identities was fairer to authors, 44% agreed whereas only 30% disagreed. That respondents should reject open identities despite seeing its ethical advantages probably relates to their views on a final question, whether reviewers would be less likely to deliver strong criticisms were their identities known to authors. Sixty-five percent of respondents thought that open reviewer identities would lead to less strong criticisms. While some perhaps thought this a positive (in that unduly harsh criticisms would be tempered), others undoubtedly were reflecting common concerns that open identities will diminish review by compromising reviewers. As one respondent put it “if a junior scientist is asked to review a document by a more senior scientist, one who may at some point in her/his career become her/his supervisor/boss, she/he might be inclined not [to] be too harsh” (#3187, 35–44, Earth and Environmental Sciences, editor/author/reviewer). A related concern is that reviewers who do not pull their punches shall suffer retribution from vengeful colleagues later. To date studies [20, 32, 33] have failed to show if reviews under open identities are any less critical. The extent to which reviewers have suffered retribution from offended authors is unknown at present. However, perceptions are hugely important here—especially where they may disincentivize reviewers. Hence, not only is more research required into how open identities affects the reviews themselves, but a more precise investigation of attitudes to open identities is necessary to help separate facts from perceptions and better inform researchers about the advantages and disadvantages of open identities peer review. This is especially so since, in addition to the fact that researchers do seem to see ethical worth in open identities, incentivizing reviews through making them citable products rely upon reviewers committing their names to these products.

Amongst the alternatives proposed by respondents when invited to leave free-text comments, many argued that if reduction of bias is the goal, that double-blind review (where not only are reviewers anonymous to authors, but authors’ identities are withheld from reviewers) is the optimal option: “Reviews should be double blind. Knowing the name of authors influences the quality of review process and introduces bias” (#2957, 35–44, Agriculture and Food Sciences, author). Amongst those who saw the value of open identities yet remained skeptical of its potential drawbacks, a good compromise was seen to be making this option voluntary: “Open identities in review would be intimidating to many. The best approach is volunteer open identity. Open identities in review would be intimidating to many. The best approach is volunteer open identity” (#4332, 55–64, Earth and Environmental Sciences, editor/author/reviewer), or enabling pseudonymity for reviewers: "A pseudonym system ensures that reviewers can be objective without fear of retribution” (#7325, 35–44, Biology and Life Sciences, editor/author/reviewer).

### Open reports

Respondents were very favourable towards publishing review reports alongside research outputs, with around 3 in 5 believing this will improve peer review and only a fifth thinking that it will make it worse. Almost 2 out of 3 believe review reports provide useful information for the reader. As one respondent reported: “For me, crucially in open peer review is that the review reports are open in the end. This allows readers to verify what criticism has been outed during the review process. One can verify if reviewers were rather positive or negative and maybe if one reviewer was even rejecting the paper, but still the editor made another decision” (#3104, 25–34, Earth and Environmental Sciences, author/reviewer). Other respondents emphasised the pedagogic value of open reports, e.g., “Open reports are hugely beneficial for young scientists because it has allowed me to see what the review process is like [and …] to see what kinds of things are acceptable to request as a reviewer and how detailed my review should be” (#4127, 25–34 Earth and Environmental Sciences author/reviewer).

Three out of five, meanwhile, think that publishing reports will increase review quality. This perception is interesting, but whether or not open reports actually lead to improvements in review quality remains an open question. What evidence there is on this question, is inconclusive: van Rooyen et al.’s 2010 empirical study of the reviews themselves [33], found no improvement in quality, while in Elsevier’s currently ongoing pilot study with open reports, for which interim results are available, a third of editors reported an improvement in the overall quality of review reports. On a less positive note, respondents to our survey thought that publishing reviews might lead to less strong criticisms (46% agreeing) and disinhibit reviewers (52% agreeing). Our finding chimes with Taylor & Francis Group’s survey [22] whose reviewers similarly thought open reports would deter reviewers, but not with Elsevier’s open reports pilot where more than 9 in 10 reviewers who declined to take part said their decision was not influenced by the open review. Why should open reports potentially deter reviewers? In addition to simply not wishing to publish their reviews, it may be that some believe that making reviews suitable for publication will take longer and hence make each review more time-intensive. Van Rooyen et al. [33] found that this is indeed the case. However, more than three thirds of the respondents to Nicholson and Alperin’s small survey thought it would take no extra effort or only moderate extra effort to make their peer reviews suitable for public posting [28].

### Open participation

Just over half of the respondents think allowing the wider community to contribute to the review process will improve peer review, versus 28% who believe it will make it worse. Similar numbers agreed that that “close circles of reviewers and editors hold back innovative research” (47%) and that all those with sufficient knowledge should be able to review, regardless of background (45%). This last point divided opinions sharply, however, with almost as many (38%) expressing disagreement. This sharp division of opinions is reflective of strong beliefs on both sides, with strong opposition between those who think opening participation can resolve possible conflicts associated with editorial selection of reviewers (e.g., biases, closed-networks, elitism) and possibly improve the reliability of peer review by increasing the number of reviewers on the one hand, and on the other, those who see open participation as opening the gates to unqualified reviewers whose credentials and motives remain opaque. Perhaps the most fundamental question for open participation remains, however, whether people will take it upon themselves to review voluntarily. Our respondents agreed heavily (85%) with the commonsense proposition that reviewers are more likely to review if they are invited. As one respondent commented: “My skepticism centres around the fact that Open Peer review sounds to me like open comments on blogs newspapers etc. which generally attract a set of commenters who enjoy commenting not necessarily commenters who are qualified to comment” (#2548, 35–44 Social Sciences author/publisher).

### Open interaction

Our respondents were heavily in favour of open interaction–peer review where direct reciprocal discussion between author(s) and reviewers, and/or between reviewers, is allowed and encouraged–with more than two thirds of respondents believing it will improve peer review and a scant 16% thinking it will make it worse. Faced with the statement that “increased interaction between authors and reviewers will result in better publications”, respondents were equally emphatic in their approbation: more than three quarters agreed, while fewer than one in ten disagreed. Given that in comparison to some other more radical elements of OPR, open interaction presents a fairly incremental change, this level of support suggests that this is presently a likely route for journals wishing to experiment with “open” peer review processes. However, as a reviewer of this paper has helpfully pointed out, although such systems might be welcomed by researchers, such interactivity would first need to be supported technologically by manuscript-handling platforms, but that functionality will only be developed when a critical mass of journals request it. Moreover, journals may be reticent as they fear such interactivity may increase the time and editorial overhead required for peer-review.

### Open pre-review manuscripts

Respondents were almost evenly split on the question of whether manuscripts being made immediately available (e.g., via pre-print servers like arXiv or bioRxiv) in advance of any formal peer review procedures would improve peer review, with 36% against and 41% in favour. However, on the question of whether pre-review manuscripts *should* be made available in this way, 45% were against while less than a third (32%) were in favour. This view seems to be consistent across all research disciplines. A majority from the social sciences (54.5%), mathematics and statistics (62.1%), law and political sciences (60%), computer sciences / IT (60.8%) and other disciplines (52%) believe open manuscripts would improve peer review, while the disciplines that believe it would actually make peer review worse are agriculture and food sciences (62.8%) and health sciences (51.4%), chemistry (43.4%). Hence, although the plurality of respondents see*potential* for improving peer review through open pre-review manuscripts, the plurality is nonetheless against actually making manuscripts openly available. That the results should be reversed in this way is somewhat puzzling, but probably suggests that while respondents saw the advantages of open manuscripts for peer review, they believed that advantage to be outweighed by other disadvantages, such as the dilution of the literature, e.g., “not sure about making papers available before peer review. What if the paper contains important mistakes but is already read by a large number of people before the reviews are made? [The] [r]eview process is also a filter to prevent bad quality papers to “pollute” the bibliographical research of scientists” (#5480, 25–34, Earth and Environmental Sciences, author/reviewer). Another possible concern was that open pre-review manuscripts lead to the danger of being “scooped” (e.g., “This will lead to innovative papers by small groups being “scooped” by large groups with greater resources”, #8274, over 64, Biology and Life Sciences, editor/author/reviewer). However, others argued that immediate publication actually protects against scooping (e.g., “I think all manuscripts should initially be deposited on non-commercial preprint servers [… to] prevent authors from getting unfairly scooped, because an invited reviewer slows down the review process on purpose”, #7812, 55–64, Biology and Life Sciences, editor/author/reviewer).

### Open final-version commenting

Respondents were strongly in favour of allowing readers to review or comment on final “version of record” publications via comment sections, blog articles, online journal clubs or social media, with 55% for and just 16% against. But despite this, only a minority considers such commenting to be a formal part of peer review (29% agreeing and 51% disagreeing). This suggests that respondents still see peer review as a publication sub-process, with a core aim being to vet and improve manuscripts for publication, rather than as an ongoing phenomenon which continues after publication.

### Open platforms

Respondents tended to be more in favour than not of the proposition that open platforms (de-coupled review that is facilitated by a different organizational entity than the venue of publication) would improve peer review. However, it must be noted that more than half of all those who responded to this question either proclaimed themselves neutral (23.8%) or said they didn’t know (27.7%), indicating that this element is either poorly understood, or that respondents found its effects difficult to judge.This is perhaps reflective of the fact that ‘open platforms’ is a fringe OPR trait [17], which is perhaps ‘open’ in a different sense to the other traits discussed here, aiming not so much at transparency or participation, but rather at increasing the efficiency of the peer review process.

## Conclusion

The results of this cross-disciplinary survey, which received 3,062 full responses, shows largely positive, but varied, attitudes towards OPR amongst authors, reviewers and editors. The majority (60.3%) of respondents believe that OPR as a general concept should be mainstream scholarly practice (although attitudes to individual traits varied, and open identities peer review was not generally favoured), as they also are for other areas of Open Science, like Open Access and Open Data. Younger generations were particularly in favour. We also observe surprisingly high levels of experience with OPR amongst our sample: three out of four (76.2%) of our respondents said that they had they had taken part in an OPR process as either author or reviewer or editor (although note again our provisos regarding the ways in which our sample may be skewed).

There were high levels of support for most of the traits of OPR, particularly open interaction, open reports and final version-commenting. The high level of support for open interaction between reviewers and authors, a nominally secondary trait of OPR, should be investigated further. As this is, in some respects, an incremental change to peer review processes, this might be the first option for those journals looking to experiment with aspects of OPR.

However, there seems to be a rather strong pushback against open identities, with 50.8% (1461 of 2878 respondents who expressed an opinion) believing that open identities will make peer review either worse or much worse. It is remarkable that open identities be the least favoured trait, as it is the OPR trait most commonly included in definitions (present in 110 of 122 definitions studied). Respondents see value in open identities but believe it could potentially open particularly younger researchers to consequences from aggrieved authors or lead to the dilution of criticism. Empirical research is needed to establish whether these concerns are legitimate—what is the prevalence of such reprisals from aggrieved authors? Are the criticisms in peer reviews conducted under open identities actually any more or less strong? In the meantime, one recommendation may be for those publishing outlets interested in implementing open identities to make it optional so that reviewers are able to choose whether or not to make their identities open. Taken together, these findings are very encouraging for OPR’s prospects for moving mainstream. However, more research is needed. OPR is an evolving phenomenon and hence future studies are to be encouraged, especially to further explore differences between disciplines and monitor the evolution of attitudes. Specific areas for further investigation into attitudes to OPR include:

- Are these findings of levels of experience with and attitudes towards OPR consistent across studies?
- Which specific OPR systems (run via journals or third-party services) do users most prefer?
- What measures might further incentivize the uptake of OPR?
- How fixed are attitudes to the various facets of OPR and how might they be changed?
- What are attitudes to OPR for research outputs other than journal articles (e.g., data, software, conference submissions, project proposals)?
- How have attitudes changed over time? As OPR gains familiarity amongst researchers and is further adopted in scholarly publishing, do attitudes towards specific elements like open identities change? In which ways?
- To what extent are attitudes and practices regarding OPR consistent? What factors influence any discrepancies?
